# Gynoecy instability in cucumber (*Cucumis sativus* L.) is due to unequal crossover at the copy number variation-dependent *Femaleness* (*F*) locus

**DOI:** 10.1038/s41438-020-0251-2

**Published:** 2020-03-15

**Authors:** Zheng Li, Yonghua Han, Huanhuan Niu, Yuhui Wang, Biao Jiang, Yiqun Weng

**Affiliations:** 1 Horticulture Department, University of Wisconsin, Madison, WI 53706 USA; 2 College of Horticulture, Northwest A&F University, Yangling, Shaanxi 712100 China; 3 Institute of Integrative Plant Biology, School of Life Sciences, Jiangsu Normal University, Xuzhou, 221116 China; 4 Vegetable Research Institute, Guangdong Academy of Agricultural Science, Guangzhou, Guangdong 510640 China; 5 USDA-ARS, Vegetable Crops Research Unit, Madison, WI 53706 USA

**Keywords:** Plant breeding, Plant molecular biology

## Abstract

Cucumber, *Cucumis sativus* is an important vegetable crop, and gynoecy has played a critical role in yield increase of hybrid cucumber production. Cucumber has a unique genetic system for gynoecious sex expression, which is determined by the copy number variation (CNV)-based, dominant, and dosage-dependent *femaleness* (*F*) locus. However, this gynoecy expression system seems unstable since monecious plants could often be found in *F*-dependent gynoecious cucumber inbreds. We hypothesized that gynoecy instability (gynoecy loss) may be due to unequal crossing over (UCO) during meiosis among repeat units of the CNV. In this study, using high throughput genome resequencing, fiber-FISH and genomic qPCR analyses, we first confirmed and refined the structure of the *F* locus, which was a CNV of a 30.2-kb tandem repeat. Gynoecious plants contained three genes: *CsACS1*, *CsACS1G*, and *CsMYB*, of which *CsACS1G* is a duplication of *CsACS1* but with a recombinant distal promoter that may contribute to gynoecy sex expression. In two large populations from self-pollinated gynoecious inbred lines, ‘gynoecy loss’ mutants were identified with similar mutation rates (~0.12%). We show that these monecious mutants have lost *CsACS1G*. In addition, we identified gynoecious lines in natural populations that carry two copies of *CSACS1G*. We proposed a model to explain gynoecy instability in *F*-dependent cucumbers, which is caused by UCO among *CSACS1/G* units during meiosis. The findings present a convincing case that the phenotypic variation of an economically important trait is associated with the dynamic changes of copy numbers at the *F* locus. This work also has important implications in cucumber breeding.

## Introduction

The common ancestor of all angiosperms was likely hermaphroditic with perfect flowers that have both stamens and pistils^1^. During the evolution of flowering plants, sex determination is an important mechanism to increase genetic fitness by promoting outcrossing, and decreasing inbreeding^2^. One such strategy taken by species in the Cucurbitaceae family is monoecy in which plants produce unisexual flowers on the same individual. Among the ~950 species in this family, ~50% are monecious^3,4^ including the economically important vegetable crop cucumber, *Cucumis sativus* L. that has long been a favorite model for study of sex determination in plants.

Three types of flowers can be present in a cucumber plant: staminate (male), pistillate (female) and hermaphrodite (bisexual/perfect). By default, all floral buds contain staminate and pistillate primordia at early stages of development; selective arrest of either staminate or pistillate flower development results in female or male flowers, respectively, and no abortion of either staminate or pistillate primordia allows development of hermaphroditic flowers^5–9^. Extensive studies have been conducted on the genetic and physiological bases on sex determination in cucumber. Early studies have established three genes, *F* (*femaleness*), *m* (*andromonoecy*), and *a* (*androecy*), and their interplays in sex determination in cucumber^5,6,10–17^. Thus, a cucumber plant may be monecious (*MMffAA*, with both male and female flowers), gynoecious (*MMFFAA* or *MMFFaa*, with only female flowers), subgynoecious (*MMFfAA* or *MMFfaa*, with few male flowers in the beginning nodes and all female flowers later), andromonoecious (*mmffAA* with bisexual flowers and male flowers), hermaphroditic (*mmFFAA*, or *mmFFaa*, with only perfect flowers), or androecious (*MMffaa* or *mmffaa* with only male flowers). Sex expression in cucumber could also be modified by other genes or quantitative trait loci (QTL), as well as environmental factors such as temperature, and photoperiod^6,14–16,18–22^.

The wild (*C. s*. var. *hardwickii*), semi-wild (*C. s*. var. *xishuangbannesis*) cucumbers and most landraces of cultivated cucumber (*C. s*. var. *sativus*) are monecious. However, from the cucumber breeding perspective, gynoecious sex expression has the obvious advantage for the increase of fruit yield. Tkachenko^12^ was probably the first to study the inheritance of gynoecious sex expression in cucumber who reported that ‘femaleness’ in a Japanese variety was governed by a pair of genes with ‘femaleness’ being dominant to ‘maleness’. The monogenic segregation was confirmed by Galun^6^ and Shifriss^13^ who designated the genetic factor as *Acr* for accelerating the differentiation of pistillate flowers in gynoecious lines, which was later renamed as the *F* (*femalenes*s) locus. The dominant allele (*F*) functions to increase the percentages of female flowers in a dosage-dependent manner^13,23^.

In 1950s, the identification of gynoecious lines from Japanese and Korean materials (for example, PI 220860), and discovery of methods for sex conversion^24–26^ expedited the incorporation of gynoecy into commercial F_1_ hybrid cucumber production^27,28^, and understanding of the genetic and physiological basis of gynoecious sex expression in cucumber. Before the cloning of the *F* locus, it has been well established that the phytohormone ethylene is the major regulator of cucumber sex determination^29–32^. There is a high level of correlation between *F* locus-dependent femaleness and elevated endogenous levels of ethylene; inhibition of ethylene biosynthesis increases the staminate tendency^30,33^. Indeed, it was found that all three main sex determination genes, *M*, *F* and *A*, are members of the *aminocyclopropane-1-carboxylic acid synthase* (*ACS*) gene families (*CsACS1G* for *F*; *CsACS2* for *M*, and *CsACS11* for *A*) catalyzing the rate-limiting step in ethylene biosynthesis^18,34–37^. Trebish et al.^34^ were the first to link *CsACS1* with the *F* locus: they found that while monecious (*ff*) cucumbers possess a single *CsACS1* gene, gynoecious ones (*FF*) have an additional copy of *CsACS1*, *CsACS1G*, which co-segregates with the *F* locus; *CsACS1* and *CsACS1G* have different distal promoter sequences that may be responsible for the exclusive expression of *CsACS1G* in *FF* (gynoecious) and *Ff* (subgynoecious) plants^35^. Knopf and Trebitsh^38^ further found that the promoter region of *CsACS1G* was the result of a recombination between *CsACS1* and a *branched-chain amino acid transaminase* (*BCAT*) gene (exons 8, 9, and 10). Recently, during a genome-wide survey of the cucumber genome for structural variation, it was found that the *F* locus was due to copy number variation (CNV) of a 30.2-kb region with monecious and gynoecious lines carrying one and two copies, respectively^39^. However, a detailed description of the CNV and the structure of the *F* locus is still lacking.

CNV refers to the rearrangements of DNA segments which typically are larger than 1 kb, resulting in the loss or gain of these genomic sequences^40^. CNVs are widely distributed in the genomes of many organisms and have been extensively studied in the human genome due to their association with numerous diseases^40–43^. CNV is also ubiquitously present in plant genomes and associated with many traits of biological and agricultural importance, such as flowering time in Arabidopsis and wheat^44–46^, *Rhg1*-conferred cyst nematode resistance in soybean (SCN)^47–50^, grain size in rice^51^, aluminum tolerance in maize or boron tolerance in barley^52,53^, and herbicide resistance in the weed palmer amaranth (*Amaranthus palmeri*)^54,55^.

CNV is a key contributor to genetic variation. Tandem gene clusters of multigene families in particular are rearrangement hotspots and a major source of novel gene formation^56–58^. CNVs seem to form at a faster rate than other types of mutation, and abiotic stresses may increase the speed CNV^40^. For example, in Arabidopsis, CNVs could be observed among individuals separated by only one generation; numerous CNVs affecting hundreds of genes had already originated after only five generations^59–61^. In cucumber, Osipowski et al.^62^ reported 626 CNVs among three individual plants derived from a common ancestor after 21 or 22 generations of continuous self-pollination. CNVs could be generated through different genetic mechanisms including nonallelic homologous recombination (NAHR) or unequal crossing over (UCO), which results from aberrant homology recognition during homology-based DNA repair or meiosis^61,63^.

In cucumber, gynoecy instability is a common phenomenon in which monecious plants are found at low frequencies in highly inbred, *F* locus-dependent gynoecious populations. Varying numbers of *CsACS1G* copies could also be found in the same gynoecious inbred line^39^ (see below). We hypothesize that the monecious lines found in gynoecious progeny (‘gynoecy loss’) may be caused by loss of the *CsACS1G* copy at the *F* locus due to UCO during meiosis. Thus, the objectives of this study were triple folds. (1) Clarify the CNV structure of the *F* locus governing gynoecious sex expression in cucumber; (2) Examine the scope of CNV at the *F* locus in natural cucumber populations; and (3) Investigate the association of ‘gynoecy loss’/instability with UCO at the *F* locus. We first verified the CNV associated with the *F* locus with fiber-FISH, genome re-sequencing, and quantitative real-time PCR (qPCR) using genomic DNA as templates. In large populations of two gynoecious inbred lines (Gy14 and G06), we identified monecious ‘gynoecy loss’ mutants and confirmed the gynoecy instability was due to elimination of the *CsACS1G* copy through UCO during meiosis. The flow chart and reasoning of our work is summarized in Supplemental file 1 (Fig. S1)

## Results

### *F* locus-dependent gynoecy in Gy14 cucumber is associated with a 30-kb tandem repeat

**Fig. 1:**
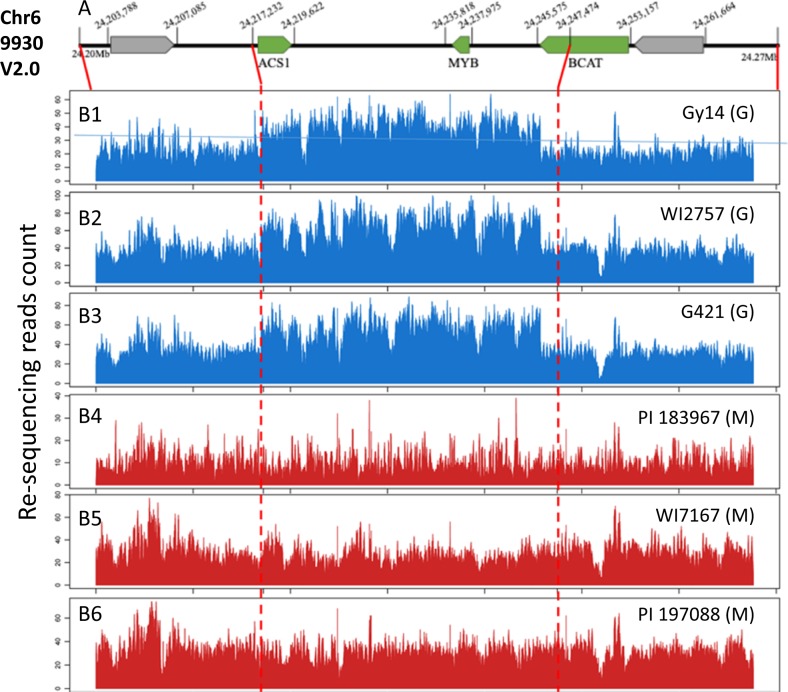
Copy number variation at the F locus in cucumber. **a** This region has ~30 kb in the monecious line 9930 with three predicted genes (coordinates based on V2.0). **b** Alignment of re-sequencing reads against the 30-kb region reveals an extra copy in gynoecious (G) lines (B1–B3), but not in monecious (M) lines (B4–B6). Vertical red dotted lines delimit the CNV region

The gynoecious Gy14 is a US pickling type inbred line while the monecious 9930 is a North China type (Chinese Long). Draft genomes of both lines have been developed. Previous studies found that, as compared with monecious cucumbers, gynoecious ones possess an extra copy of *CsACS1* with a recombinant distal promoter (*CsACS1G*), and the 30.2 kb sequences were duplicated which constitutes the *F* locus^38,39^. We manually annotated this 30.2 kb region in the 9930v2.0 assembly and predicted three genes: *CsACS1*, *CsMYB*, and *CsBACT* (Fig. 1a). Then, using 50 kb of this region in 9930v2.0 (30 kb plus 10 kb up-stream and down-stream sequences) as the reference, we mapped Illumina re-sequencing reads from six cucumber lines including three gynoecious cucumbers Gy14, G421, and WI2757 that are knownly carrying the *F* locus, and three monecious ones, PI 183967 (wild cucumber, *C. s*. var. *hardwickii*), WI7167 (semi-wild cucumber, *C. s*. var. *xishuangbannesis*), and PI 190788 (a landrace from India). Each genome was sequenced at 15–30 × depth of coverage. As compared with the flanking 10 kb regions, the average number of reads was nearly twice as many in the 30-kb region in all three gynoecious lines (Fig. 1B1–B3), but no such difference was observed in the three monecious lines (Fig. 1B4–B6) supporting early notion that the 30.2-kb region was duplicated in gynoecious cucumbers.

**Fig. 2:**
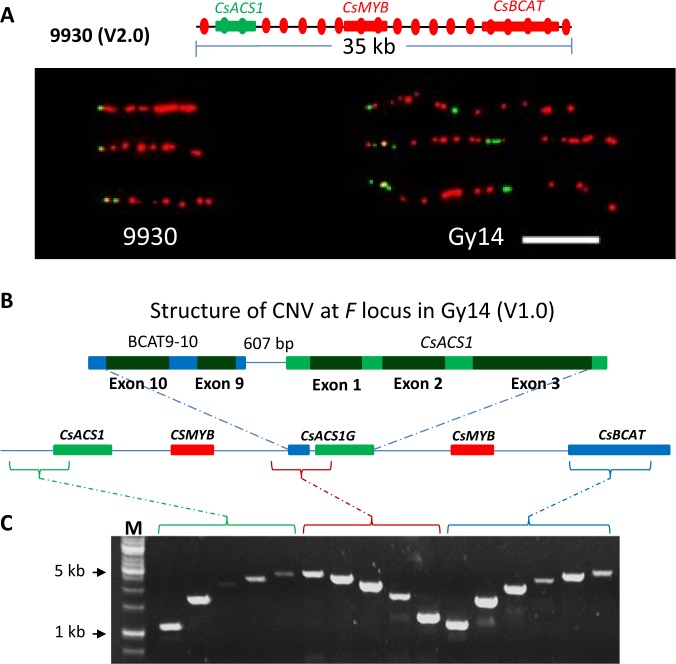
Structure of *F* locus in gynoecious Gy14 cucumber. **a** Fiber FISH analysis reveals a tandem duplicate of 30-kb region in Gy14 as compared with the monecious 9930 cucumber. The green signals are from probes of *CsACS1* genomic DNA sequence, and the red signals are from pooled 17 probes from other sequences in the 30-kb region (up). The approximate targeting positions of 19 probes are shown as green and red oval spots. Three representative DNA fibers of Gy14 and 9930 are shown (down). Bar = 10 µm. **b** Genomic structure of the *F* locus in Gy14. The exons 9 and 10 of the *CsBCAT* gene is part of the second copy of *CsACS1*, which are collectively called *CsACS1G*. **c** One-end anchored PCR is used to analyze the continuity of the *F* locus in the 5’ end (green), 3′ end (blue), and ‘junction point) (brown). M = DNA size ladder

To examine the orientation of this duplication, we conducted fiber FISH using single copy DNA sequences from ~40 kb region of 9930 V2.0 as probes. Among 19 probes designed [one probe every 2 kb; Fig. 2a; Supplementary File 2 (Table S1)], two (FISH-2 and FISH-3) were located in the *CsACS1* region, and the rest were in up-stream (one probe) or down-stream (16 probes) of *CsACS1*. The two *CsACS1* probes were pooled and labeled with the green dye, and the remaining 17 probes were pooled and labeled with the red dye in FISH of both 9930 and Gy14 genomic DNA fibers. The distribution of FISH signals on the DNA fibers at the *F*-locus region is shown in Fig. 2a. As expected, in 9930, the relative positions of red and green signals were consistent with its genome structure, while in the gynoecious Gy14, the physical length of labeled DNA fiber was twice that of 9930, and the green and red signal patterns suggested a tandem duplication of the 30.2 kb region in Gy14.

### Genomic structure of *F* locus in Gy14 and 9930 cucumbers

Previous studies have shown that in gynoecious cucumber plants, in addition to the non-specific *CsACS1*, the additional copy of this gene, *CsACS1G*, had a new distal promoter region (upstream from −410 bp) containing genomic DNA sequences of exons 9 and 10 of the *CsBCAT* gene due to recombination between *CsACS1* and *CsBCAT*^34,35,38^. We annotated this region in Gy14 V1.0 (~60 kb), and confirmed the existence of *CsACS1G* (BCAT exons 9 + 10 + *CsCAS1*) and *CsMYB* in the tandem repeat. Thus, in Gy14, the first copy of the 30.2 kb region was exactly the same as in 9930 (*CsACS1* *+* *CsMYB*), and the second copy was *CsACS1G* (BCAT exons 9 + 10 + *CsACS1* *+* *CsMYB*), which was followed by a complete *CsBACT* (Fig. 2b).

When this project was initiated, only Gy14 V1.0 and 9930 V2.0 assemblies were available, both of which had some gaps or N’s in this region. Thus, it was not clear if additional sequences may present in the CNV region of Gy14. We employed anchor PCR to estimate the size in target regions of the CNV in Gy14. Primers were designed at three regions for PCR including the 5′ region (upstream of *CsACS1*), the ‘junction point’ of BACT exons 9–10 and *CsACS1* (upstream of *CsACS1G*), and the 3′ region (*CsBCAT* gene) (Fig. 2c). At each location, one primer was anchored, and other primers were designed at locations such that the PCR amplicons were increased in size progressively (Table S1). The results indicated that, in each case, the amplicon sizes were consistent with expected sizes estimated from genomic sequences in Gy14 V1.0 and 9930 V2.0 (Fig. 2c) suggesting no additional large sequences are present in the CNV region in the Gy14 assembly.

**Fig. 3:**
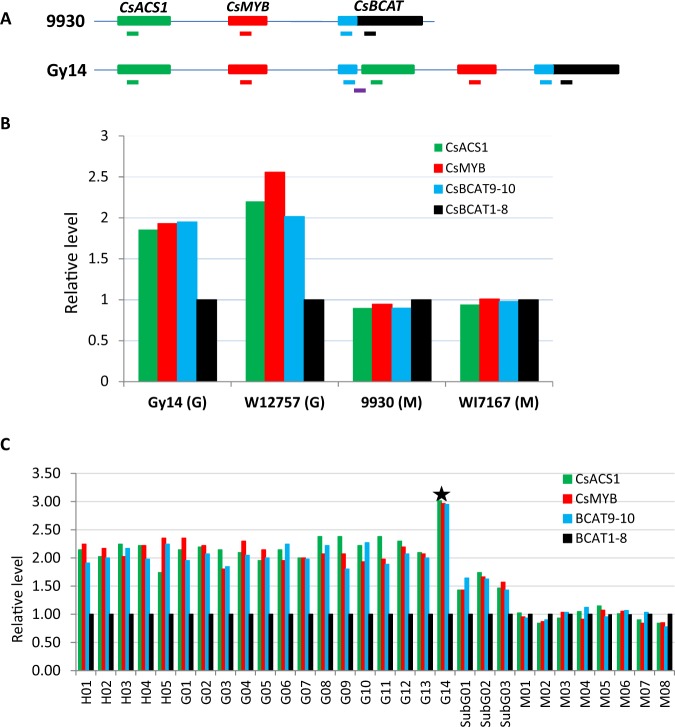
Evaluation of copy numbers of genes at the *F* locus with qPCR of genomic DNAs in cucumber. **a** Targets regions for qPCR. Green, red, blue and black horizontal bars represent genomic region of *CsACS1, CsMYB*, exons 9–10, and 1–8 of the *CsBACT* genes, respectively. Copy numbers of different regions revealed by qPCR among four representative gynoecious and monecious lines, as well as 30 additional cucumber varieties are shown in **b** and **c**, respectively. Each data point in **c** is the mean of seven replications that was normalized to that for exons 1–8 of the *CsBCAT* gene. In **c**, asterisk indicates the gynoecious line AM297 (G14) that contains three copies of the 30-kb region at *F* locus. In each line name, H = hermaphroditic, G = gynoecious, M = monecious. Asterisk indicates the gynoecious line AM297, of which CNVs value is 3

We further verified the copy numbers of individual genes in the 30.2 kb region with real-time qPCR using genomic DNA as the templates. Four pairs of gene-specific primers were designated from sequences targeting the *CsACS*1, and *CsMYB* genes, exons 9 and 10 (BACT9–10), as well as exons 1–8 (BACT1–8) of the *BCAT* gene (Fig. 3a; Table S1). The effectiveness of all primers was first confirmed with consistent electrophoretic bands from regular PCR. The qPCR with genomic DNA was performed in four representative cucumber lines: the gynoecious Gy14, and WI2757, and the monecious 9930, and WI7167. In qPCR, the level of BCAT exons 1–8 that are not involved in the CNV at the *F* locus was used as the standard to normalize the level of other three genes. The results are presented in Fig. 3b. Indeed, as compared with the adjacent genomic region, there were two copies each of the *CsACS1*, *CsMYB*, and BCAT9–10 in the 30.2 kb region of the two gynoecious lines (Gy14 and WI2757), and one copy each in 9930 and WI7167.

Previous studies^38,39^ and the results reported herein all supported ~30.2 kb duplication at the *F* locus in gynoecious cucumbers that contains two copies of *CsACS1* and BCAT exons 9 and 10. However, the exact sequences and annotation of this region have never been explicitly reported. By the time we were preparing the manuscript, the improved versions of the 9930 V3.0^64^ and Gy14 V2.0 (our unpublished data) draft genomes were just released to the public (available at https://www.cucurbitgenomics.org/). No gaps or N’s were found in the CNV region in either draft genome, which allowed verification and refinement of the results obtained in the present study. We manually annotated the CNV regions in both Gy14 and 9930, which are graphically presented in Supplementary File 3 (Fig. S2). In 9930 V3.0, the sequences from transcription start of *CsACS1* to 3′ end of *CsBCAT* is 35,572 bp. In the Gy14 V2.0 assembly, unexpectedly, two copies of *CsACS1G* (BACT9–10 + *CSACS1* *+* *CsMYB*) were annotated with a total length of 94,849 bp (Fig. S2). Since all Gy14 materials used in various studies (genome sequencing for V1.0/V2.0, and fiber FISH) were from the same source (but different generations from self-pollinations), it may hint that the triple tandem repeats of the ~30 kb region in Gy14 V2.0 may be the result of UCO (see discussion below).

We examined DNA nucleotide diversity in the *CSACS1/CsACS1G* gene region. Sequence alignment among four sequences (~4.6 kb each) including *CsACS1* from 9930 V3.0 and Gy14 V2.0, *CsACS1G* from Gy14 V2.0, and the NCBI accession DQ839406 (*CsACS1G* from gynoecious cucumber Marketmore 76F)^38^. The results are illustrated in Supplementary File 4 (Fig. S3). Sequence features for *CsACS1* (TATA box, transcription start/end, and intron/exon junctions) and *CsACS1G* (proximal and distal promoters) are also highlighted. The ~4.6 kb Gy14 and 9930 *CsACS1* genomic DNA sequences were almost identical. In addition, the sequences from −410 bp to 3′ UTR in both *CsACS1* and *CsACS1G* were also largely the same except for two SNPs (Fig. S3). These observations were consistent with previous findings with regard to sequence variation at the *F* locus in gynoecious and monecious lines^38^. The data also suggested that the duplication of *CsACS1* resulting in the *F* locus was a very recent event, and the *F* locus in the two gynoecious lines Gy14 and Marketmore 76F (DQ839406) have the same origin (see “Discussion” section below).

Knopf and Trebitsh^38^ suggested the promoter of *CsACS1G* was derived from recombination between *CsBCAT* and *CsACS1* genes. Indeed, significant sequence diversity was found in the distal promoter regions (upstream from −411 to −1700) between *CsACS1* and *CsACS1G* (Fig. S3). The sequences in *CsACS1G* from −411 upstream of transcription initiation are part of the *CsBCAT* gene which included (reverse complementary strand, in order of presence): the 8th intron (928 bp), Exon 9 (113 bp), the 9th intron (350 bp), and Exon 10 (113 bp) with a total of 1504 bp (up to −1914 from transcription start). Interestingly, the whole length of the 8th intron is 984 bp, and 928 bp was present in the distal promoter with the last 56 bp missing.

### CNV at *F* locus is associated with gynoecy expression in natural cucumber populations

Next we sought to investigate the association of CNV at the *F* locus with sex expression in natural cucumber populations. Previous studies^52,53^ and our work described above (Fig. 3b) showed that qPCR could be used to estimate copy numbers of genomic DNA sequences. We employed this method to examine the number of copies of the four sequences at the *F* locus (Fig. 3a) among 30 lines of different sex morphs including 5, 14, 8, and 3 of hermaphroditic (*FF*), gynoecious (*FF*), monecious (*ff*), and subgynoecious (SubG, *Ff*) cucumber lines, respectively (Supplementary File 5 or Table S2). The three SubG lines were F_1_ from gynoecious × monecious crosses that were heterozygous at the *F* locus. The qPCR results among the 30 samples are illustrated in Fig. 3c. All monecious lines had one copy each of the three sequences (*CsACS1*, *CsMYB*, and BACT9–10), and all hermaphroditic and gynoecious lines except AM297 (G14 in Fig. 3c) had two, and the three SubG F_1_ hybrids had 1.5 copies. These results were consistent with two copies of the 30-kb unit in gynoecious plants containing the *F* locus.

**Fig. 4:**
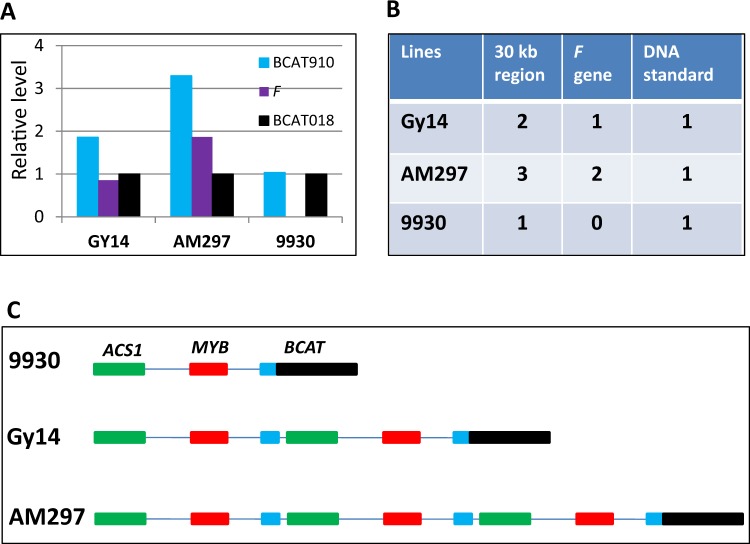
Triple tandem repeats of *CsACS1/CsMYB* at *F* locus in gynoecious cucumber line AM297. **a** and **b** copy number of different regions of the *F* locus in Gy14, 9930, and AM297 revealed by genomic DNA qPCR. **c** Diagrams of the *F* locus structure in the three cucumber lines. The purple bars indicate copy numbers of the ‘junction region’

Among the 14 gynoecious varieties examined, AM297 was an interesting exception that had three copies of (*CsACS1* + *CsMYB* + BACT9–10) sequences. To clarify the structure of the *F* locus in AM297, we conducted genomic DNA qPCR among Gy14, 9930 and AM297 using primers from BCAT9–10, BCAT1–8, and a new primer pair, F-gDNA, which targeted the ‘junction point’ in the unique promoter region of *CsACS1G* (Fig. 3a). The left and right primers of F-gDNA were near the 10th exon of *BCAT*, and the start of *CsACS1*, respectively. Because of the unique design of the primer pair, there would be no amplicon in the monecious 9930 cucumber due to opposite directions of the two primers while the expected amplicon size would be 111 bp in Gy14 (Table S1). The qPCR results (Fig. 4a) suggested that AM297 carries triple tandem repeats of the ~30 kb region with two copies of *CsACS1G* (Fig. 4b). The structures of the *F* locus in the three varieties are summarized in Fig. 4c, in which AM297 was predicted to have the same triple repeat as in Gy14 V2.0 (Fig. S2).

All the gynoecious lines carried at least one copy of *CsACS1G* suggesting they may have a single origin. Indeed, all the 14 gynoecious lines we used (Table S2) belong to US pickling or slicing market groups or the mini (Beit Alpha) cucumbers with rather recent breeding histories. To confirm this, we examined sequence diversity in the 50-kb genomic region around *CsACS1* region among 48 re-sequenced cucumber varieties including 10 gynoecious lines, which are listed in Supplementary File 6 (Table S3). From the re-sequencing data, 68 SNPs and 3 InDels were detected (Table S3), and were used to construct a phylogenetic tree (Supplementary File 7/Fig. S4). All 10 gynoecious varieties were in the same clade, and the grouping of monecious lines was based largely on their taxonomic status or geographic origins. This observation supported the common origin of the *F* gene in all gynoecious lines.

### Gynoecy instability may be due to loss of *CsACS1G* through UCO among tandem repeats of the CNV at the *F* locus

**Fig. 5:**
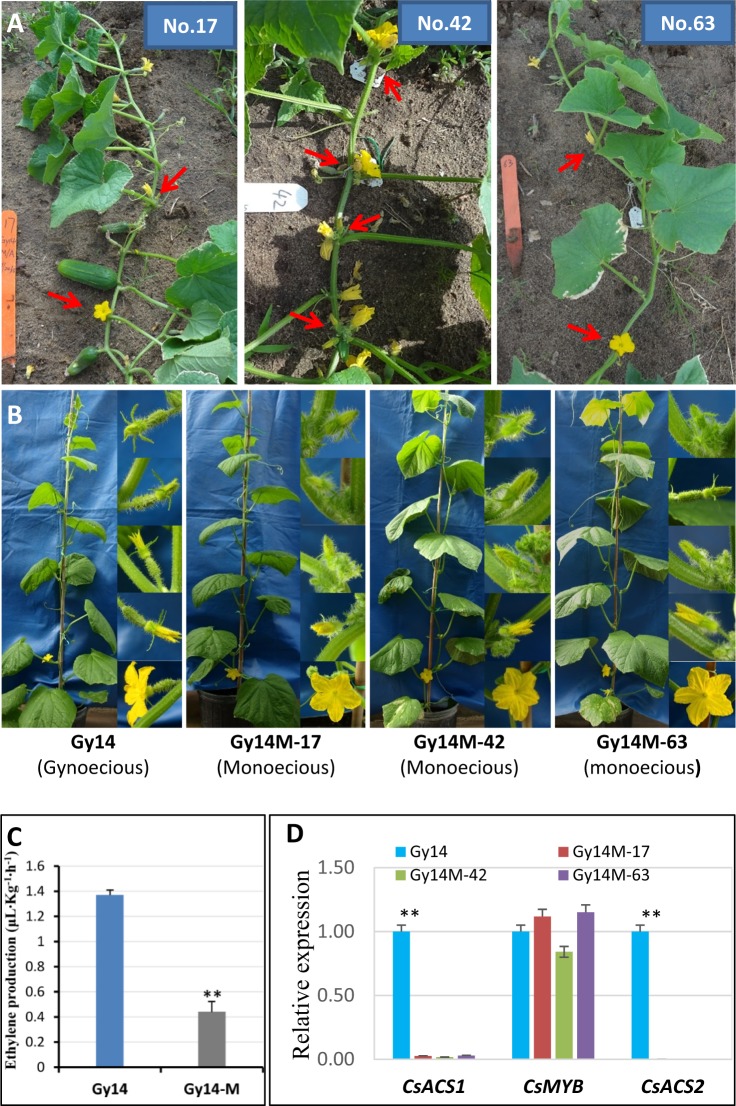
Characterization of ‘gynoecy loss’ lines in Gy14 cucumber. **a** Three representative ‘gynoecy-loss’ mutant lines (Nos. 17, 42, and 63) identified in the field among ~2,400 Gy14 gynoecious plants (red arrows show some male flowers). **b** Segregation of monecious plants among selfed progeny of three mutant plants (gynoecious Gy14 to the left as control). **c** Comparison of ethylene release rate between Gy14 and Gy14-M (mean of three mutant lines). **d**. Comparison of expression level of two genes (*CsACS1* and *CsMYB*) in the *F* locus and *CsACS2* in Gy14 and three monecious mutant lines. Error bars represent the SD from three biological replicates, and asterisks (**) indicate significant differences at *P* *<* 0.01

In cucumber, monecious plants could be found at low frequency among self-pollinated progeny of highly inbred gynoecious lines (‘gynoecy loss’). Varying copies of *CsACS1G* may present in gynoecious lines (Fig. 3c). We hypothesize that ‘gynoecy loss’ may be the result of UCO among the 30-kb repeat units at the *F* locus. To prove this, in 2015 field season, we grew 2236 gynoecious Gy14 plants in an open field. All these plants were derived from a single Gy14 plant through two generations of self-pollinations. Throughout the growing season, plants with at least one male flower were tagged. By end of the season, 66 such plants were identified, but only three exhibited consistent subgynoecious sex expression, which were designated as No. 17, 42, and 63, respectively (Fig. 5a). Thus, the mutation (‘gynoecy loss’) rate was 0.13% in Gy14 (3/2236). In 2017, we conducted a similar experiment using another gynoecious line G06 that belongs to the Mediterranean (Beit alpha) type cucumber. Among 2573 gynoecious G06 plants grown in greenhouse conditions, three subgynoecious plants (G06M-B, G06M-C, and G06M-E) were identified. The mutant frequency of ‘gynoecy loss’ in G06 was 0.11%, which was very close to 0.13% observed in Gy14 suggesting gynoecy instability is a common phenomenon in *F*-dependent gynoecious cucumbers regardless of the genetic backgrounds.

The three subgynoecious mutant plants from Gy14 were self-pollinated, and the progeny were observed for sex expression. The segregating sex phenotypes (gynoecious, subgynoecious, and monecious) in their progeny populations suggested the three lines were true ‘gynoecy loss’ mutations. We further self-pollinated the monecious individuals, and the offspring were all monecious in the following six generations of self-pollinations, which were designated as Gy14M-17, Gy14M-42, and Gy14M-63, respectively (Fig. 5b), indicating these ‘gynoecy loss’ mutations were stable. We re-sequenced the three mutant lines with >15× coverage, and the reads were aligned against the 30-kb *F* locus region of the 9930 V2.0 draft genome. We found the same depth of coverage of reads in the 30-kb region as the flanking regions (Supplementary File 8/Fig. S5B2-B4) suggesting the extra copy of *CsACS1* in Gy14 (i.e., *CsACS1G*; Fig. S5B1) have lost in the three monecious mutant lines. We also conducted qPCR using primers in the *F* locus to examine copy numbers of target genes at the *F* locus in the three ‘gynoecy loss’ lines, which further supported the loss of *CsACSG1* in these mutants (Supplementary File 9/Fig. S6).

UCO among repeat units at the *F* locus occurs in gynoecious (G) plants would generate two types of gametes: gametes with duplication (1 *CsACS1* *+* 2 *CsACS1G* alleles) and gametes with deletion (1 *CsACS1*). The three ‘gynoecy loss’ mutants (Gy14M-17, −42 and −63) might represent the products from deletion of *CsACS1G*. While we did not try to identify products from duplication events because the large amount of work to screen gynoecious plants. Alternatively, AM297 with two copies of *CsACS1G* might represent such product from duplication of the 30-kb repeat of UCO. Thus, we investigated the inheritance of sex expression by examining lines and derived segregating populations which carry different copies of *CsACS1G* (Table 1). It is known that the *F* gene in cucumber has a dosage effect, and the F_1_ from gynoecious (*FF*) × monecious (*ff*) cross is subgynoecious (*Ff*, SubG). Indeed, of 16 Gy14 × 9930 F_1_ plants observed, 15 were SubG (Table 1; Supplementary File 10/Fig. S7A, B). Similar results were obtained in F_1_ of Gy14 × XTMC (M), and Gy14 × S52 (M) crosses. In contrast, all 16 F_1_ plants from AM297 × 9930 cross were gynoecious. In addition, all the Gy14M-17/−42/−63 × 9930 F_1_ plants were monecious (Table 1; Fig. S7C, D). In the Gy14 × 9930 F_2_ population, of the 220 plants, the segregation of G:SubG:M plants was consistent with the expected 1:2:1 ratio. But among over 1000 AM297 × 9930 F_2_ plants, no SubG plants were present, and the ratio of G to M plants exhibited roughly 3:1 segregation suggesting at least two copies of the *F* gene in the AM297 haploid genome (Table 1). The no show of SubG plants in the large population also indicated that the two copies of the *F* gene were co-segregating (no recombination), which was consistent with the model structure of tandem duplication of the three 30-kb regions in AM297 predicted in Fig. 4c. These data support the hypothesis that AM297 carries two copies of *CsACS1G*, which might be derived from UCO.

**Table 1:** Segregation of sex expression in cucumber lines or populations

Lines/populations	# *CsACS1G* copies	Expected sex expression^a^	# Plants observed	# G	# of SubG	# of M	*P* for *χ*^2^ test
Gy14	2	G	2236	2170	0	3^b^	–
G06	2	G	2573	2537	0	3^b^	–
9930	0	M	20	0	0	20	–
S52	0	M	20	0	0	20	–
XTMC	0	M	20	0	0	20	–
AM297	4	G	20	20	0	0	–
(Gy14 × 9930) F1	1	SubG	16	1	15	0	–
(Gy14 × XTMC) F1	1	SubG	16	2	14	0	–
(Gy14 × S52) F1	1	SubG	16	0	16	0	–
(Gy14 × 9930) F2	0, 1, or 2	Segregating, 1G: 2 SubG: 1M	200	47	98	55	0.6976
(AM297 × 9930) F1	2	G	16	16	0	0	–
(AM297 × XTMC) F1	2	G	16	16	0	0	–
(AM297 × S52) F1	2	G	16	16	0	0	–
(AM297 × 9930) F2	0, 1, or 2	Segregating, 3G: 1M	1000	735	0	265	0.2733
(Gy14M-17 × 9930) F1	0	M	14	0	0	9	–

^a^*G* gynoecious, *SubG* Subgynoecious, *M* Monecious

^b^‘Gynoecy loss’ monecious mutants

From previous studies, it is well known that a high correlation exists between ethylene accumulation and female sex expression (see section “Introduction”). We examined ethylene releases in wild type gynoecious Gy14 and the ‘gynoecy loss’ mutants, and found significant decrease of ethylene in the three monecious mutant lines (Fig. 5c). We further examined the expression levels of *CsACS1*, *CsACS2* (*andromonoecious* or *m* locus), and *CsMYB* at the *F* locus with mRNA qPCR (Fig. 5d). Among all three mutant lines, as compared with Gy14, the expression of both *CsACS1* and *CsACS2* was significantly down regulated, whereas no significant difference in expression was found for *CsMYB* among the four lines. In our previous study, transcriptome profiling for sex expression revealed no expression difference of the *CsBCAT* gene among the three mutant lines and Gy14^65^. These data suggest that expression level of both Cs*MYB* and Cs*BCAT* genes at the *F* locus may have no direct link with gynoecy expression in cucumber.

## Discussion

### Structure and function of the *F* locus in cucumber

Cucumber has a unique system for gynoecious sex expression which is based on CNV of a 30.2 kb region containing *CsACS1G*, a duplicated copy of *CsACS1* but with recombinant promoter^38,39^. In melon and watermelon, the gynoecious sex expression is controlled by *WIP1* homologs encoding a C2H2 zinc-finger transcription factor, which is a negative regulator of femaleness^66,67^. No gynoecious *WIP1* mutant (*CsWIP1*) have been identified in natural cucumber populations. *CsWIP1* may perform a similar function in cucumber because mutations obtained by CRISPR/Cas9 (clustered regularly interspaced short palindromic repeats/CRISPR-associated protein 9) assay in this gene significantly increase the degree of femaleness^68^.

In the present study, data from fiber-FISH, analysis of resequencing reads, as well as qPCR (Figs. 1–3) all confirmed that the *F* locus for gynoecy in cucumber is a duplication of 30.2 kb region. These results were further validated with analysis of the newest 9930 (V3.0) and Gy14 (V2.0) draft genome assemblies which also, for the first time, presented a more complete fine structure of the *F* locus (Figs. S2 and S3). From previous studies^35,38,39,69–71^ and work reported herein, the following conclusions could be made. (1) The *F* locus is a duplication of 30.2 kb repeat unit. In monecious lines (*ff*), this region contains three genes: *CsACS1*, *CsMYB*, and *CsBCAT*; in gynoecious lines (*FF*), the structure of the *F* locus is *CsACS1–CsMYB–CsACS1G–CsMYB–CsBACT* (Fig. 2b). (2) There is no sequence difference between *CsACS1* and *CsACS1G* in the proximal region (+1 to −410 bp) and CDS in the gynoecious plant tested. (3) The 5′-distal promoter (upstream from −410 up to −1900) of *CsACS1G* is composed of sequences from intronic (8th and 9th) and exonic (exons 9 and 10) sequences of the *CsBCAT* gene (Fig. S3), which is very different from that in *CsACS1* supporting its origin from recombination between the *CsACS1* and Cs*BCAT* genes^38^.

The *CsACS1G* sequences from Gy14 (a US pickling cucumber) and Marketmore 76F (A US slicing cucumber) are identical (Fig. S3). This is expected because the *F* locus in both gynoecious lines were probably from the same source, MSU713–5, which, in turn, was introduced from the Korean cucumber line ‘Shogoin’ (PI 220860)^25^. In fact, most modern gynoecious cucumbers in commercial production may share the same source of *F* locus-dependent gynoecy from MSU713-5^28,72,73^. This can explain the clustering of all gynoecious lines in one clade (Fig. S4) which would otherwise be clustered based on their geographic origins if genome-wide molecular markers were used in phylogenetic analysis^74,75^.

The effects of the duplication at the *F* locus on the expression of *CsACS1* and *CsACS1G* in gynoecious and monecious cucumbers have been investigated in several studies. First, the recombination between *CsBACT* and *CsACS1* resulting in the distal promoter of *CsACS1G*, which introduced a putative open-reading frame (ORF) (from position 528 of Gy14 *CSACS1G* or position 666 in DQ839406_ACS1G.1, Fig. S2), but Shiber et al.^71^ did not detect any transcripts from this putative ORF thus ruling out the possibility that its product may be the *F* locus. In gynoecious plants (*FF*), the transcript level of *CsACS1/G* is much higher^38,65^. On the other hand. knock-down of *CsACS1G* expression in gynoecious cucumber resulted in monoecy^71^. We found that, in ‘gynoecy loss’ mutants (loss of *CsACS1G*), both ethylene level and expression of *CsACS1* were significantly reduced as compared with the wild type (Fig. 5d). These observations suggested the higher expression of *CsACS1/G* in gynoecious plants is directly linked with gynoecium development.

Among the three genes involved in the CNV at this *F* locus (Figs. 3b and S2), the expression of *CsMYB* and *CsBACT* did not seem to be affected by the duplication event in the generation of the *F* locus. No significant differences in expression of the two genes were identified between monecious and gynoecious cucumbers^65^ (also Fig. 5d). This is in contrast with the *Rhg1* locus of soybean. *Rhg1*-conferred cyst nematode resistance in soybean^47–50^ in which the SCN resistance level is positively correlated with copy numbers of a 31.2 kb repeat unit. The simultaneous upregulated expression of all three genes in the repeat unit is required for the resistance^47^. It is interesting to see why *CsMYB* expression remains unchanged in gynoecious cucumbers.

### Gynoecy instability and CNV dynamics in gynoecious cucumbers

In cucumber, the *F* locus-dependent gynoecy is relatively stable. However, it is common that monecious plants are found at low frequency in the self-pollinated progeny of gynoecious inbred lines which we call ‘gynoecy loss’. Indeed, in two gynoecious lines, the US pickling cucumber Gy14 (Fig. 5) and the mini cucumber inbred G06, we found 0.13% and 0.11% plants that were monecious ‘gynoecy loss’ mutants. We show that these mutant lines have lost the *CsACS1G* copy at the *F* locus (Table 1, Fig. S5). Since the *F* locus contains two copies of the 30.2 kb repeat, these data suggested that gynoecy expression is not stable due probably to the dynamic change of copy numbers of the 30-kb unit.

Such dynamic change of copy numbers is common at CNV loci in plant and animal genomes, which is most likely caused by NAHR or UCO^63,76^. UCO depends on misalignment of adjacent homologous sequences. The result of UCO is deletion or duplication/triplication of a genomic sequence, which is likely to form a CNV locus. A CNV locus with tandem repeat structure, or located in adjacent position in the same chromosome, may have the potential to take place further UCO^57,76^. One good example for phenotype-associated CNV instability due to UCO is the *Rhg1* locus for SCN resistance in soybean. The level of *Rhg1*-conferred SCN resistance level in soybean is positively correlated with copy numbers of a 31.2 kb repeat unit^47^. While there is an extensive diversity in copy numbers among different resistant lines, significant variation of copy numbers and disease resistance level was also observed within the same variety^50^. It has been shown that copy number at *Rhg1* is unstable within a released variety over a relatively small number of generations due to NAHR or UCO^47–50^. NAHR has also been shown to be responsible for CNV of different alleles at the *I* (inhibitor) locus in soybean for the colors of seed coat^77^, and many other CNV-based phenotypic variation^78–80^.

**Fig. 6:**
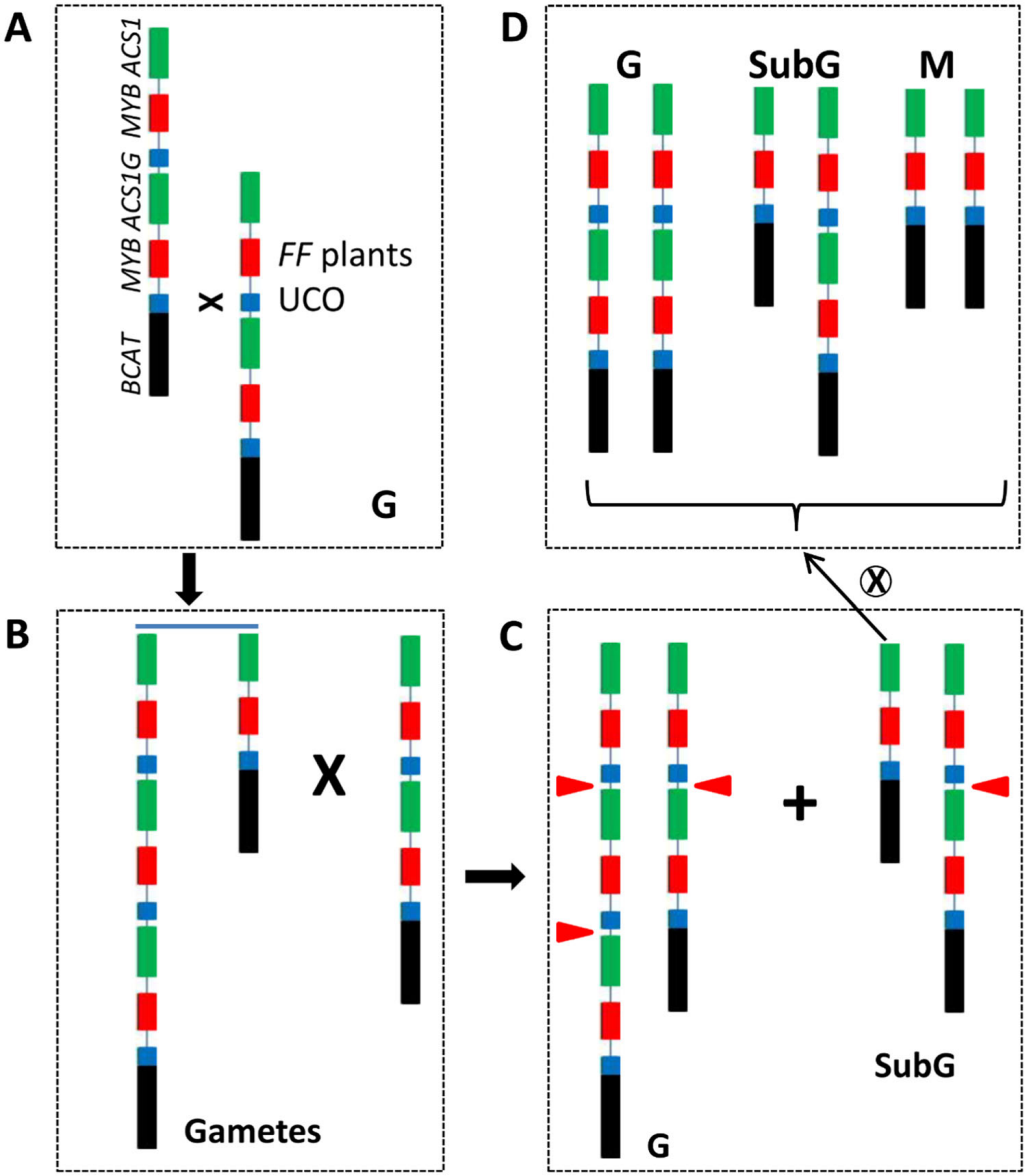
A model explaining generation of ‘gynoecious loss’ monecious mutants from F gene-based gynoecious cucumber plants through unequal crossing over (UCO). **a** At meiosis, UCO between the two copies results in two types of gametes with single or triple copies of the 30-kb region. **b** Self-pollination among gynoecious plants carrying the two types of gametes produces gynoecious (G, three *F* genes) and subgynoecious (SubG, equivalent of *Ff* heterozygotes) plants. **c** Self-pollinated progeny of SubG plants will segregate for G, SubG, and monecious (M) plants. **d** The illustrated genomic structure is same as in Fig. 3a. Red triangles show the *F* gene

UCO may also explain the gynoecy instability or ‘gynoecy loss’ observed in the present study. For easy description, a model is provided in Fig. 6 to explain UCO events resulting in ‘gynoecy loss’ monecious mutations in Gy14 cucumber. During meiosis, the two 30.2 kb repeat units containing three genes misalign and subsequent UCO (Fig. 6a) would result in gametes with deletion and duplication of *CsACS1G* sequences (Fig. 6b). In self-pollination, the union of these gametes with normal *F* gametes will result in gynoecious (G) and subgynoecious (SubG) plants (Fig. 6c). Self-pollination of SubG individuals will segregate for G, SubG, and monecious (M) plants (Fig. 6d). The M plants were confirmed by the observation of no sex expression segregation in the following self-pollination (Fig. 5b). The mutation rate for the M plants from UCO is likely low (0.13% in Gy14). We did not genotype G plants carrying two or more *CsACS1G* copies in the field due to the sheer large numbers. The presence of CNV = 3 (two copies of *CsACS1G*) plants could be indirectly evidenced from the identification of AM297 in natural cucumber populations (Fig. 4), and in two copies of *CsACS1G* in Gy14 V2.0 (Supplementary Fig. S2). In fiber FISH, the hybridization patterns in multiple gynoecious lines also indicated possible varying copies # in the *F* locus (data not shown).

It is puzzling why the CNV has 30.2 kb as the repeat unit in which two of the three genes (*CsMYB* and *BCAT*) have no difference in expression level before (in monecious) and after (in gynoecious) the duplication. Interestingly, after examining several draft genomes (https://cucurbitgenomics.org/ and https://www.ncbi.nlm.nih.gov/), we found high degree of micro-synteny of the *ACS1*–*MYB*–*BCAT* trio in both gene order and content across multiple cucurbit crops, which also extends to as far as Arabidopsis. It is not known if the triplet genes are required for gamete fertility or any other fitness advantages during evolution.

## Materials and methods

### Identification and phenotypic characterization of ‘gynoecy loss’ mutants

Two gynoecious cucumber inbred lines Gy14 and G06 (genotype *FF* at the *F* locus) were used to test our hypothesis on ‘gynoecy loss’, which belong to the North American pickling and Mediterranean beta alpha (mini) market group, respectively. Both lines have undergone at least six generations of self-pollination. In 2015 field season, 2500 Gy14 plants were grown in the field plot at the University of Wisconsin Madison Hancock Agriculture Research Station (HARS, Hancock, WI, USA). In 2017 spring, a similar work was conducted with G06, in which sex expression was examined among over 3000 plants in greenhouses at the Jingyang Agriculture Research Station (Xianyang, Shaanxi, China). At reproductive stage, each plant in both experiments was examined for sex expression. All plants with one or more male flowers were tagged. Subgynoecious plants were self-pollinated. Multiple plants from the selfed progeny were planted in the glasshouses for observation of sex expression. Selected mutants were self-pollinated to observe segregation among the progeny.

The gynoecious cucumber inbred line AM297 (aka, ‘Telegraph Improved’) was found to contain three copies of *CsACS1/G* gene the *F* locus in haploid genome. Both Gy14 and AM297 were crossed with three monecious lines 9930, XinTaiMiCi (North China type), and S52 (South China type). In addition, the three ‘gynoecy loss’ (monecious) mutant lines (Gy14M-17/-42/-63) were crossed with 9930. Sex expression of these F_1_ plants were recorded in the greenhouses. In addition, F_2_ plants were produced from AM297 × 9930 F_1_ and Gy14 × 9930 F_1_ for observation of segregation of sex expression.

Selected cucumber varieties (total 37, Supplementary Table S2) were also grown at HARS in multiple seasons for observation of sex expression according to Li et al.^81^. Leave samples of these lines were collected for analysis of copy numbers for genes at the *F* locus.

### Copy number estimation of F locus with fiber fluorescence in situ hybridization (FISH) and resequencing reads alignment

For visual access of the copy numbers at the *F* locus, fiber FISH was conducted with Gy14 and 9930 inbred lines. Based on the 9930 V2.0 genome assembly (https://cucurbitgenomics.org/), 19 primer pairs were designed in the targeting ~40.0 kb region of single copy DNA sequences. The locations of each probe in different versions of Gy14 and 9930 genome assemblies are listed in Supplementary Table S1. The resulting PCR amplicons (average length ~2 kb) were used as probes.

FISH procedures on genomic DNA fibers were described previously^82,83^. The probe labeling was performed according to published protocols^83^. DNA probes were labeled with digoxigenin-dUTP or biotin-dUTP via nick translation and detected with anti-dioxigenin antibody coupled with Rhodamine (Roche) or anti-avidin antibody conjugated with FITC (Vector Laboratories), respectively. Hybridization signals in two-color fiber-FISH were detected with a three-layer antibody detection system as described^84^. Images were captured digitally using a CCD camera, and the final image adjustments were done with Adobe Photoshop v6.0.

We estimated CNV at the *F* locus from resequencing data of nine cucumber lines with different sex morphs including the gynoecious Gy14, G421, and WI2757, monecious lines WI7167, PI 183967, PI 197088, and the three Gy14 ‘gynoecy loss’ mutant lines (Gy14M-17/-42/-63). The genome of each line was re-sequenced with Illumina Hi-Seq 2000 sequencing platform at >15× coverage. Sequencing reads of different mutant lines were aligned against the 40 kb *F* gene region of the 9930 V2.0 draft genome using BWA pipeline following Pan et al.^85^.

### Genome structure analysis at *F* locus

The draft genomes of *F* locus region in both 9930 and Gy14 were manually annotated. To verify the structure of *CsACS1G*, we amplified the 5′ and 3′ regions as well as the *CsACS1*–BCAT ‘junction point’ with progressive PCR in which one end of the amplicon was anchored and the other end was at different locations. The primer sequences and their positions are listed in Table S1. All amplicons in the junction point were confirmed with sub-cloning and Sanger sequencing.

The structure of the *F* locus by evaluating copy numbers of genes in the CNV region was confirmed with real-time quantitative PCR (qPCR) using genomic DNA as the template. The primers were designed from different regions of selected genes (Table S1). All PCR reactions were conducted in a 96-well plate using the ABI 7500 Fast Real-Time PCR System (Applied Biosystems, CA, USA) with the SYBR green qPCR master mix (Bio-Rad, Hercules, CA, USA). The amplification was initiated by heating to 95 °C for 10 min, followed by 40 cycles at 95 °C for 20 s and 65 °C for 30 s. Each result was derived from seven independent replications.

The genomic DNA sequence diversity of the 50 kb *F* locus was examined in 48 re-sequenced cucumbers lines reported previously^86,87^ and the three ‘gynoecy loss’ mutants. SNPs within the target region were called with the BWA-GATK4.0 workflow using 9930 V2.0 as the reference following Wang et al.^88^. All detected polymorphisms (SNPs or InDels) in the 30-kb CNV sequence in the ‘gynoecy loss’ mutants were verified by PCR and Sanger sequencing. These polymorphic markers in the 50 kb region were employed to construct a phylogenetic tree among the 48 cucumber lines to understand the origin of the *F* locus. Sequence alignment and clustering were performed with MEGA 7.0 (http://www.megasoftware.net/) using the maximum likelihood method with 1000 bootstrap replications.

The new versions of draft genomes for both Gy14 (V2.0) (our unpublished data) and 9930 (V3.0)^64^ were recently released (https://cucurbitgenomics.org/). We compared the 30-kb CNV region in 9930 with that of Gy14 to verify results from other experiments in this study.

### Expression of sex determination-related genes with mRNA qPCR

We examined expression level of *CsACS1*, *CsMYB* genes in the CNV region as well as the andromonoecy (*m*) gene *CsACS2* in relation to sex expression. The apices of Gy14, 9930 and three ‘gynoecy loss’ mutants were harvested for RNA extraction and first-strand cDNA synthesis^89^ and qPCR using the procedure described above with the cucumber *actin2* as the reference to normalize the expression data (primer info is provided in Table S1). Each sample was run with three biological and at least three technical replicates with appropriate statistical (*t* tests) analysis of the data.

### Measurement of ethylene release

To measure the ethylene production rate, shoot apices were excised from seedlings at the fourth true leaf stage. Equal amount of samples of the three mutant lines was mixed as ‘Gy14-M’. There were three biological replicates from both Gy14 and Gy14-M with each replicate containing shoot apices from 15 plants. Each sample was placed in a 12 ml container and sealed with a rubber stopper. After incubation at 25 °C for 14 h in the dark, 1 ml of gas was withdrawn using a gas-tight syringe from the headspace, which was analyzed using the Trace GC Ultra gas chromatography system (Thermo Scientific, USA) that was equipped with a flame-ionization detector and a capillary column for ethylene measurement. The instrument was calibrated with an ethylene gas standard, and the amount of ethylene released from shoot apices per 1 kg fresh weight and per hour was calculated. All determinations were made in triplicate.

## Supplementary information

Supplemental files
