# Supplementary material for: Gynoecy instability in cucumber (Cucumis sativus L.) is due to unequal crossover at the copy number variation-dependent Femaleness (F) locus
Source: Hortic Res. 2020 Mar 15;7:32. doi: 10.1038/s41438-020-0251-2 (PMC7072070; doi:10.1038/s41438-020-0251-2)

Supplementary File 1 (Fig. S1). Flow chart of work of the present study.

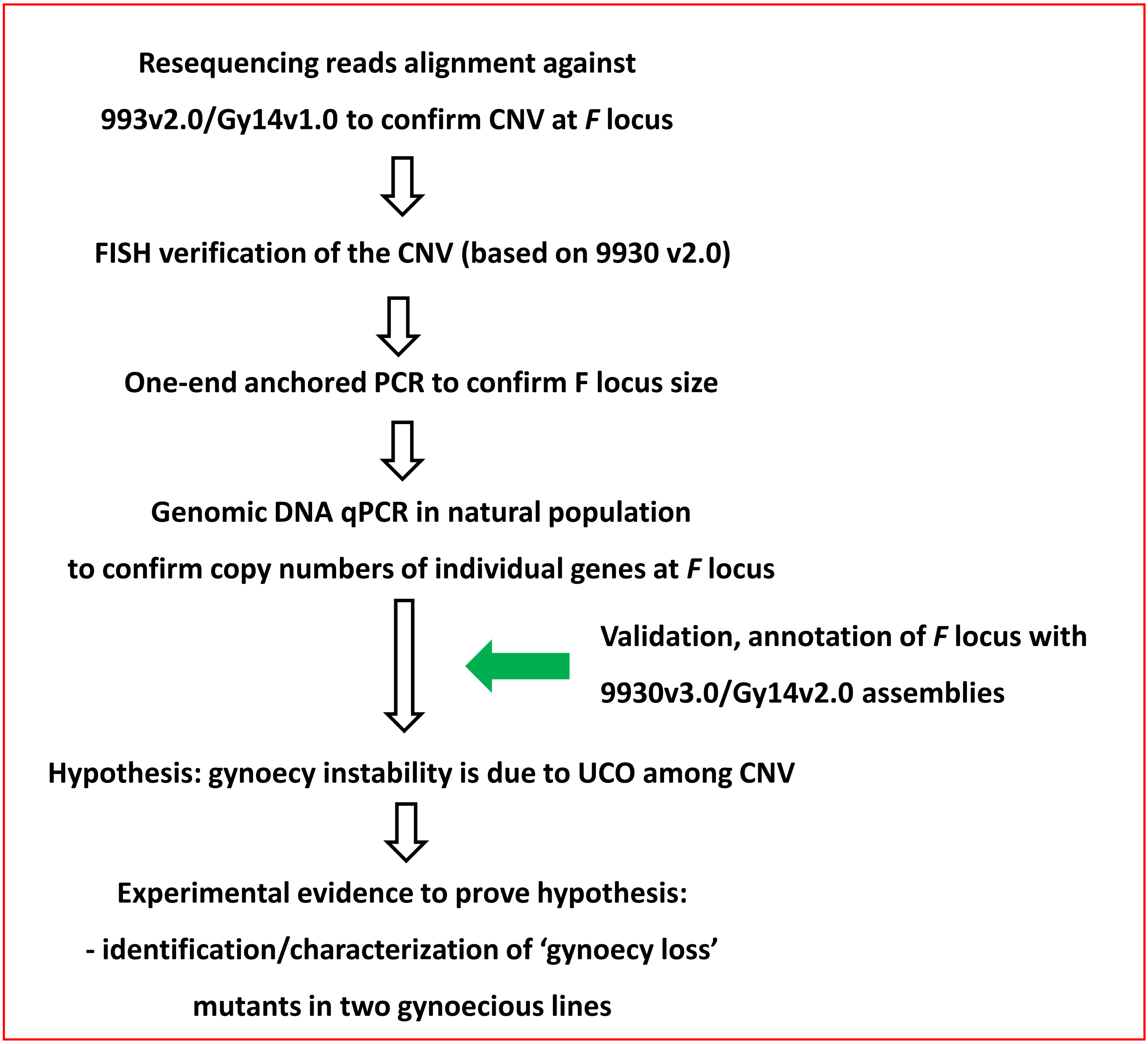

Supplementary File 2 (Table S1). Information of primers used in this study.

Primer names	Forward primer (5′ to 3′)	Chromosome	9930 V2.0	9930 V3.0	Gy14 V2.0	Reverse primer (5′ to 3′)	9930 V2.0	9930 V3.0	Gy14 V2.0
**Fiber FISH probes**									
FISH-01	TATGTCCCTACGCCCTAT	Chr6	24,215,158	26,239,898	27,486,394	CATGATACATTTACCCACA	24,216,862	26,241,602	No hit
FISH-02	CCCTCCTGTCCCTCTATT	Chr6	24,217,202	26,241,914	27,488,444	CCAAACTTCCTCGTCTTC	24,218,852	26,243,564	27,490,093
FISH-03	AGTAACGAAGACGAGGAA	Chr6	24,218,829	26,243,541	27,490,070	AAAGGTTGGAGGAAAGAA	24,220,593	26,245,317	27,491,848
FISH-04	TTTCGGTATCTTCCTATG	Chr6	24,220,946	26,245,670	27,492,201	TCTCCCTCACCCTATTTT	24,222,677	26,247,409	27,493,940
FISH-05	TTTACCGTTGATGCTCGTC	Chr6	24,222,941	26,247,648	27,494,215	AGGTCTAATGGGTGATAG	24,224,814	26,249,564	27,496,088
FISH-06	ATTAGACCTATTCAACCCAT	Chr6	24,224,806	26,249,556	27,496,080	CAAGAAACATATCCTGCA	24,226,799	26,251,411	27,497,934
FISH-07	TCATTTCTGGGCGTTCTT	Chr6	24,226,841	26,251,453	27,497,976	TCCTTTCATTTCGGCTCT	24,228,592	26,253,204	27,499,726
FISH-08	AAAGAAAGTGAATCAAGGTT	Chr6	24,228,794	26,253,406	27,499,928	CAATGAAGGGAAAAGAAA	24,230,740	26,255,351	27,501,865
FISH-09	TTGATTGGAAGGAGAAGC	Chr6	24,230,997	26,255,608	27,502,122	CAACTCCATAACGGGTCA	24,232,547	26,257,170	27,503,684
FISH-10	TTTATTACTTAGTTGTGCTTAC	Chr6	24,232,682	26,257,305	27,503,819	ATGCTGATAGTTATCGGA	24,234,668	26,259,291	27,505,805
FISH-11	CATCCCTTCTCATTGTAA	Chr6	24,234,865	26,259,488	27,506,002	TGTTTTCGTTCTTTTCAC	24,236,554	26,261,177	27,507,690
FISH-12	TCCTCCACTAACCCATTC	Chr6	24,236,874	26,261,497	27,508,010	TTTCCCTTCTTCCTGACT	24,238,436	26,263,059	27,509,570
FISH-13	CTGTTTTGAATCGGTCAG	Chr6	24,238,574	26,263,197	27,509,708	CCTTTTCCTTTCATTACC	24,240,466	26,265,089	27,511,599
FISH-14	GAAGAAGGAAGGAGGAAT	Chr6	24,240,721	26,265,344	27,511,847	TCTCACAAATGAAAGGGA	24,242,490	26,267,113	27,513,655
FISH-15	GATAATGAAGCAATACCC	Chr6	24,242,435	26,267,058	27,513,600	AAGTTCGACCAATAAGTT	24,244,342	26,268,963	27,515,501
FISH-16	AAGGGTACTAACTTATTGG	Chr6	24,244,316	26,268,937	27,515,475	AATTGATGTGATGGCTGA	24,246,112	26,270,699	27,517,178
FISH-17	CAAATGAATACGGGAACA	Chr6	24,246,313	26,270,900	27,517,379	TTACTTCAGGTGCGATGC	24,248,153	26,272,740	27,578,997
FISH-18	GAAGATAGGGCAGGCAACG	Chr6	24,248,349	26,272,936	27,579,193	TTGGGAACTATTTCAAGGTTTG	24,249,976	26,274,563	27,580,820
FISH-19	ACCTGAGATGCAAGAGCA	Chr6	24,250,090	26,274,677	27,580,934	CTTTTGACCGAACCCTGA	24,251,893	26,276,480	27,582,737
**Anchor PCR, CNV, 5' end (left)**									
Anchor-L1	AGCTTAAACACGTAGGTATGAATAAA	Chr6	24,213,203	26,237,942	27,484,439	TGGTGTTGAATCAACCACACTTTA	24,214,158	26,238,894	27,485,395
Anchor-L2		Chr6				TAACATATCAAAGGCTCATGTCGT	24,215,208	26,239,948	27,486,444
Anchor-L3		Chr6				TAAAAGGAATTGGAACCTAAATCT	24,216,223	26,240,963	27,487,481
Anchor-L4		Chr6				TCTGCCATTTTCTTCTTTCACCTTA	24,217,064	26,241,804	27,488,334
Anchor-L5		Chr6				TAATTACTTGATTCTCGGCAAGAC	24,217,991	26,242,703	27,489,232
**Anchor PCR, CNV, CsACS1-BCAT junction point**									
Anchor-JP1	TTTTAAGATCCAGGTGTCCTTTGAG	Chr6	24,243,275	26,267,896	27,514,434	AATGCGGGTAAGCCGTGGT	24,218,222	26,242,934	27,489,463
Anchor-JP2	GATTAGTCAAGATGTTGGTGGTTGG	Chr6	24,244,215	26,268,836	27,515,374				
Anchor-JP3	CGATGTTCGTTCCTTCCTTGATATA	Chr6	24,245,335	26,269,922	27,516,400				
Anchor-JP4	TCAAAAACAAATGAATACGGGAACA	Chr6	24,246,306	26,270,893	27,517,372				
Anchor-JP5	TCAAACTGGCCTATTTTCCTTTCTT	Chr6	24,247,284	26,271,871	27,518,344				
**Anchor PCR, CNV, 3' end**									
Anchor-R1	AGATGAAGGTTTTGCGTTGAAGTAT	Chr6	24,245,068	26,269,655	27,516,135	TTACCAGATTACAAATGGGGCTTA	24,246,031	26,270,618	27,517,097
Anchor-R2		Chr6				CTGACAATTTAGCTTTGGACGGTA	24,247,026	26,271,613	27,518,087
Anchor-R3		Chr6				TACAACCTGAATCTTCCGAACAGT	24,248,022	26,272,609	27,578,866
Anchor-R4		Chr6				AAAAGCAAAAGGCTATTCTGATGT	24,249,012	26,273,599	27,579,856
Anchor-R5		Chr6				ATGTTTCACCTGTTGGGAACTATT	24,249,988	26,274,575	27,580,832
Anchor-R6		Chr6				TTGACCATTGGATTGGAACAGAA	24,251,012	26,275,599	27,581,856
**Sequencing, *CsACS1G* promoter**									
CsACS1G-seq	TCAAAAACAAATGAATACGGGAACA	Chr6	24,246,306	26,270,893	27,517,372	AATGCGGGTAAGCCGTGGT	24,218,222	26,242,934	27,489,463
**qPCR for copy # of genomic DNA**									
CsACS1g-gDNA	AAGGCATTGGTAGAATTTATGGCGG	Chr6	24,218,227	26,242,939	27,489,468	TGGATAGTATGGAGTTGGGAGGA	24,218,388	26,243,100	27,489,629
CsMYB-gDNA	TAGATTGATGAGCAAAAGCAGGAGC	Chr6	24,236,713	26,261,336	27,507,849	TATGGGATGATGCCAAGCAGTAGT	24,236,863	26,261,486	27,507,999
CsBCAT-gDNA1 (Exons 9 and 10)	AAATCTCTTGGGACTGGCAGAAAAG	Chr6	24,245,913	26,270,500	27,516,979	AAATGGTGTTGGGGTTGTTTCACA	24,246,072	26,270,659	27,517,138
BCAT-gDNA2 (Exons 1-8)	TCACTGCTTCCACAAACTGATCAAC	Chr6	24,251,141	26,275,728	27,581,985	TGAGGGTCTAAAGGCGTACAGAA	24,251,280	26,275,867	27,582,124
F-gDNA (CsACS1G juntion point)	CGGACGGGCATAGTTATGAGC	Chr6	24,247,441	26,272,028	27,518,501	AGTGTCTCCGACCAGAGCAGGTA	24,217,355	26,242,067	27,488,597
**qPCR for mRNA expression**									
CsACS1-mRNA	GGGTCTTGCCGAGAATCAACTAT	Chr6	24,217,966	26,269,922	27,489,207	GGGTAAGCCGTGGTAATCTTGG	24,218,217	26,242,929	27,489,458
CsMYB-mRNA	TCGGAGAAGCCTCCATGTTTG	Chr6	24,237,529	26,262,152	27,508,665	GCCTTGCCTCAAAAAATAGGTCTT	24,237,712	26,262,355	27,508,868
CsACS2	CCTTACTATCCTGGATTTGACAGAG	Chr1	21,939,051	no hit	no hit	AATGTCTTCGATTGTGGAGCGTTC	21,939,944	12,940,823	12,959,445
CsActin2 (reference)	ATTCTTGCATCTCTAAGTACCTTCC	Chr6	22,425,592	24,457,766	25,695,808	CCAACTAAAGGGAATAACTCACC	22,425,839	24,458,013	25,696,055

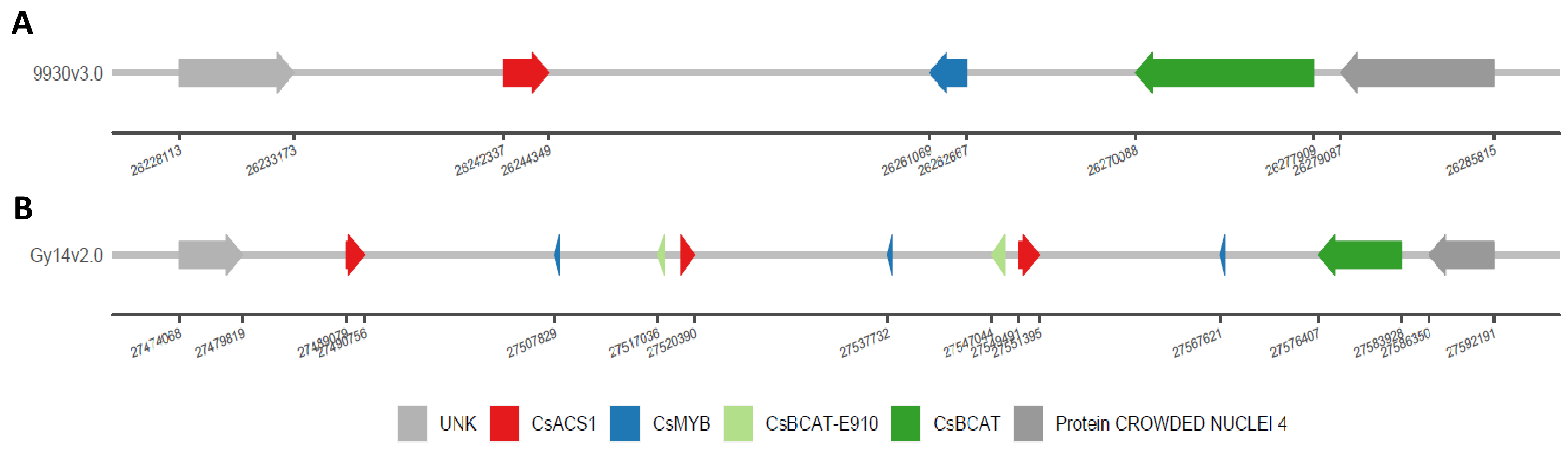

Supplementary File 3 (Figure S2). Annotation of the *F/f* locus in 9930 V3.0 (up) and Gy14 V2.0 (down) region. The *CsACS1* region in monoecious 9930 spans ~35.5 kb harboring three predicted genes (*CsACS1*, *CsMYB* and *CsBCAT*) which is ~94.8 kb in Gy14 V2.0 that is predicted to have three tandem repeats including one copy of *CsACS1*, and two copies of *CsACS1G*. Each *CsACS1G* consists of a recombinant promoter from BCAT exons 9 and 10, and *CsACS1*. Note the orientations of each gene are different.

Supplementary File 4 (Figure S3). Alignment of genomic DNA sequences of *CsACS1* from 9930 and Gy14, and *CsACS1G* from Gy14 and a gynoecious line (Marketmore 76F, DQ839406) reported in Knopf and Trebitsh[38]. The annotated regions in 9930 *CsACS1* are highlighted in yellow following Trebitsh et al[34].

```
9930_ACS1                AATAATAATAAGAACAAGAACAACAAAAAAGGCTGTTTTGGATGGAAAAAACTATTTTTT 60
Gy14_ACS1                -----------GAACAAGAACAACAAAAAAGGCTGTTTTGGATGGAAAAAACTATTTTTT 49
Gy14_ACS1G               ------------------------------------------------------------ 0
DQ839406_ACS1G.1         AAAGGTAAACGATGATTCTACAAAATCACCCTATGCCTACGATAGACTGACGGTCCAATT 60


9930_ACS1                CTTCTTATTTTTGGGTGGTAAA---CGTAGAATATTGAATAATATAATGAATTGATTTTG 117
Gy14_ACS1                CTTCTTATTTTTGGGTGGTAAA---CGTAGAATATTGAATAATATAATGAATTGATTTTG 106
Gy14_ACS1G               ------------------------------------------------------------ 0
DQ839406_ACS1G.1         CATTTTATCTTCGATAAGTCCCATTTGTAATCTGGTAAGTACAGAATAGAGTTGTTGTGA 120


9930_ACS1                AAAATACTTACCA-------------------------ACCCCTAAAGGGAAATTAGGTG 152
Gy14_ACS1                AAAATACTTACCA-------------------------ACCCCTAAAGGGAAATTAGGTG 141
Gy14_ACS1G               -------------------TCCATAAGCAATCCTGCAAAAATTCAGCCATCACATCAATTG 42
DQ839406_ACS1G.1         AACAACCCCAACACCATTTCCATAAGCAATCCTGCAAAAATTCAGCCATCACATCAATTG 180
                                                               *   *       * ** *  **
                                      Begin BCAT Exon 10 (reverse)
9930_ACS1                TGTGTGTATAGAGTATAGAGAG--------ACATGAAGTAGGGTGTTTCTCCGGCCGACG 204
Gy14_ACS1                TGTGTGTATAGAGTATAGAGAG--------ACATGAAGTAGGGTGTTTCTCCGGCCGACG 193
Gy14_ACS1G               TAAGGAAAATGCTTCAAAAACCACACGTCCATATTATTGCCCTTACTCGAGCAACCCACG 102
DQ839406_ACS1G.1         TAAGGAAAATGCTTCAAAAACCACACGTCCATATTATTGCCCTTACTCGAGCAACCCACG 240
                         *  *   *  *  *  * *           *  ** *       *  *    *  ** ***

9930_ACS1                GACATAAACTTTTGATATGAGACCTGTTTTGTCCAACCTTTTCAGCCTTTTACAAAGTGT 264
Gy14_ACS1                GACATAAACTTTTGATATGAGACCTGTTTTGTCCAACCTTTTCAGCCTTTTACAAAGTGT 253
Gy14_ACS1G               TAAAAAGAGTGGAGATATAAGCTCAATCTAGTCAAACGATTTCCCATTTCCAAGACCACC 162
DQ839406_ACS1G.1         TAAAAAGAGTGGAGATATAAGCTCAATCTAGTCAAACGATTTCCCATTTCCAAGACCACC 300
                          * * * * *   ***** **  *  * * *** ***  ****    **  *  *

9930_ACS1                GTTTGTTGCTTTGATATGGACGGGTTTTAGACTACAGGTGGAAATTAAGATCCTACTTAT 324
Gy14_ACS1                GTTTGTTGCTTTGATATGGACGGGTTTTAGACTACAGGTGGAAATTAAGATCCTACTTAT 313
Gy14_ACS1G               TAGTGA--------TGATGAATGGTTTGTGTTGAAGAGACTTTAATACCATTCTACGTAT 214
DQ839406_ACS1G.1         TAGTGA--------TGATGAATGGTTTGTGTTGAAGAGACTTTAATACCATTCTACGTAT 352
                            **         *   **  *****  *   *   *     * **  ** **** ***
                                                    -1622(DQ839406)
9930_ACS1                GCTTTTCAATTTTATTA-ATTCATTGACGATAGATTTTACT---CTCTAATGTTTATTTG 380
Gy14_ACS1                GCTTTTCAATTTTATTA-ATTCATTGACGATAGATTTTACT---CTCTAATGTTTATTTG 369
Gy14_ACS1G               GTGTTGCAATCTCTTCTAGTTCAAAAACAAATGAATACGGGAACATGTACTTTCTTTCAA 274
DQ839406_ACS1G.1         GTGTTGCAATCTCTTCTAGTTCAAAAACAAATGAATACGGGAACATGTACTTTCTTTCAA 412
                         *  ** **** *  *    ****   ** *  ** *         *  ** * * * *

9930_ACS1                TCA----TCATTTGAGATTT-----------------AGGTTCCAATTCCTTTTACTCAC 419
Gy14_ACS1                TCA----TCATTTGAGATTT-----------------AGGTTCCAATTCCTTTTACTCAC 408
Gy14_ACS1G               TCAGAACTCAATTTCAATTTCCCAACTACTCATTCCACTGTTTCGCTGTCTCTCAATGTG 334
DQ839406_ACS1G.1         TCAGAACTCAATTTCAATTTCCCAACTACTCATTCCACTGTTTCGCTGTCTCTCAATGTG 472
                         ***    *** **   ****                   *** *  *  ** * * *
                                                     Auxin Responsive factor binding
9930_ACS1                TAAATATTTTAT---------------TCTTTATCCATTTAATGTTAATGTTTTCGTTAAT 464
Gy14_ACS1                TAAATATTTTAT---------------TCTTTATCCATTTAATGTTAATGTTTTCCTTAAT 453
Gy14_ACS1G               TCCAGATATTGTTTTTGTAAAATTTACCTTTTTCCGAGATATGTGACGCTGCCCACAGGT 394
DQ839406_ACS1G.1         TCCAGATATTGTTTTTGTAAAATTTACCTTTTTCCGAGATATGTGACGCTGCCCACAGGT 532
                         *  * ** *                  **** ***          *   *   *     *
                                 Start of 9th intron (reverse)
9930_ACS1                TAAATTTTCACTATTTT---------TCATCCTTTTTACACTTAAATTTAGAATTTCCC- 514
Gy14_ACS1                TAAATTTTCACTATTTT---------TCATCCTTTTTACACTTAAATTTAGAATTTCCC- 503
Gy14_ACS1G               GATATAACCACTGCTGTCCCCGTACAAAAGACCTCGTCAGCTTCAAGTAATTCATCCACT 454
DQ839406_ACS1G.1         GATATAACCACTGCTGTCCCCGTACAAAAGACCTCGTCAGCTTCAAGTAATTCATCCACT 592
                          * **   ****  * *           *  * *  *   *** ** * *    * * *

9930_ACS1                ------------TTCATAATTTCATAATTATTTTAAATTGATTGAAAGTGAGTATTATTT 562
Gy14_ACS1                ------------TTCATAATTTCATAATTATTTTAAATTGATTGAAAGTGAGTATTATTT 551
Gy14_ACS1G               GCCACAAGTCGTTCCTCGACCTGACACTCATTCAAATCAGTGCCCACTTCTCTCGTCTTT 514
DQ839406_ACS1G.1         GCCACAAGTCGTTCCTCGACCTGACACTCATTCAAATCAGTGCCCACTTCTCTCGTCTTT 652
                                     * *   *  * * * * ***  **   *     *  *   *  * ***
                         BACT Exon 9 (reverse)
```

```
9930_ACS1                AATGAAAAATCAATTGTAGA----AGTGCAA-----------------------AATTTA 595
Gy14_ACS1                AATGAAAAATCAATTGTAGA----AGTGCAA-----------------------AATTTA 584
Gy14_ACS1G               TTATCACAATCCATTATTTTACCAAGACAAACAAATAATATTCCTTACGCAGGTATTTAA 574
DQ839406_ACS1G.1         TTATCACAATCCATTATTTTACCAAGACAAACAAATAATATTCCTTACGCAGGTATTTAA 712
                              * **** *** *       **   **                       * ** *
                                   Potential ORF start (ATG reverse)
9930_ACS1                AAAACTTATATT----CGACTAAACTGAAACTGAAATTAAACTTAGGAACTAAGTCCTCC 651
Gy14_ACS1                AAAACTTATATT----CGACTAAACTGAAACTGAAATTAAACTTAGGAACTAAGTCCTCC 640
Gy14_ACS1G               ACAACGTACAAAAGAGAGAACCAACTCAATCACATTTTCGACTTACAAAACATTTGTAAA 634
DQ839406_ACS1G.1         ACAACGTACAAAAGAGAGAACCAACTCAATCACATTTTCGACTTACAAAACATTTGTAAA 772
                         * *** ** *       **   **** ** *  *  **   ***** **  *  *

9930_ACS1                TTTGCTTATAACCATTTAGAATCTTAGATTATTGTTTTGTCTTTAAAAATAGGTTTATTT 711
Gy14_ACS1                TTTGCTTATAACCATTTAGAATCTTAGATTATTGTTTTGTCTTTAAAAATAGGTTTATTT 700
Gy14_ACS1G               TATGT-TGAGATGAGATCAAATCTAAATATAGGAAATTAAATTTGAAAGTAGTTTTTTT- 692
DQ839406_ACS1G.1         TATGT-TGAGATGAGATCAAATCTAAATATAGGAAATTAAATTTGAAAGTAGTTTTTTT- 830
                         * **  *   *  *  *  ***** *   **     **   *** *** *** *** **

9930_ACS1                TCTCTTTTATTTTCATCTTTGTTGGGTAAAAGAATTGAATTAATTTAAAAAACAAAAACA 771
Gy14_ACS1                TCTCTTTTATTTTCATCTTTGTTGGGTAAAAGAATTGAATTAATTTTAAAAACAAAAATA 760
Gy14_ACS1G               -----TTAG--TACAC-CCAAGTGAA-----CACCACAATTAATCTCATAAGAGAAATTC 739
DQ839406_ACS1G.1         -----TTAG--TACAC-CCAAGTGAA-----CACCACAATTAATCTCATAAGAGAAATTC 877
                              **    * **       **        *    ******* * * **   ***
                                                                    -1594 (DQ839406)
9930_ACS1                AAATTTTAAAAACTATCTTTTAAAGTTTTCAAATTTTAACTTAGTTTTTGAAA------- 824
Gy14_ACS1                AAATTTTAAAAACTATCTTTTAAAGTTTTCAAATTTTAACTCAGTTTTTGAGAATGTTGA 820
Gy14_ACS1G               TAAGATATAAAATCCTGGTAAGAGGTCATCTTGATTGAACTCATTCTCTTCAACTCTTTT 799
DQ839406_ACS1G.1         TAAGATATAAAATCCTGGTAAGAGGTCATCTTGATTGAACTCATTCTCTTCAACTCTTTT 937
                          **  *  ****   *  *   * **  **    ** **** * * * *   *

9930_ACS1                ---------------ATGTTGATAGAGAGTAAATAACAAAATAAGA--ATTTTAAAAGTG 867
Gy14_ACS1                TAGAGGTTTTTGAGAATGTTGATAGAGAGTAAATAACAAAATAAGA--ATTTTAAAAGTG 878
Gy14_ACS1G               ATCAATCCTATAGATATTTAGAATTTGACCATTAGACCAACTCAAAATGGTTTATACCTT 859
DQ839406_ACS1G.1         ATCAATCCTATAGATATTTAGAATTTGACCATTAGACCAACTCAAAATGGTTTATACCTT 997
                                        ** * **    **  *    ** ** * * *    **** *  *

9930_ACS1                AAAGAGATGTTATTTATGATCTAATTGAGATTTTAAAAAATTTAAAACAAAAAAGT-TAT 926
Gy14_ACS1                AAAGAGATGTTATTTATTATCTAATTGAGATTTTAAAAAATTTAAAACAAAAAAGT-TAC 937
Gy14_ACS1G               TAAAAGTTGACAGTGTATAACAACTTTAA-TATTAC------AAATTCCCACCAGTTAAT 912
DQ839406_ACS1G.1         TAAAAGTTGACAGTGTATAACAACTTTAA-TATTAC------AAATTCCCACCAGTTAAT 1050
                          ** ** **  * *    * * * ** *  * ***        **  *  *  ***  *

9930_ACS1                AGAACTATTTTTAAATATTAATAAAAT-----AAACAAAACATTTACAAAATATACTAAA 981
Gy14_ACS1                ATAACTATTTTTAAATAT-AATAAAAT-----AAACAAAACAATTACAAAATATACTAAA 991
Gy14_ACS1G               AGTATTTCTTTTAATACCGTCCAAAGCTAAATTGTCAGCAAGCATGCATTTTATTGACAA 972
DQ839406_ACS1G.1         AGTATTTCTTTTAATACCGTCCAAAGCTAAATTGTCAGCAAGCATGCATTTTATTGACAA 1110
                         *  * *  ******        ***          **  *    * **   ***    **
                                                        CArG transcription regulator
9930_ACS1                ATATCAAAGTCTACAA-----TAAACAACAAATTATCAAAATAGATTAGACTATTACATG 1036
Gy14_ACS1                ATATCAAAGTCTACAA-----TAAATAACAAATTATCAAAATAGATTAGACTATTAT--G 1044
Gy14_ACS1G               CTAGACCAACCTAAGATGTTTCCTCTAAAAATATTTCAGAAATTATTATACAA-TACTAG 1031
DQ839406_ACS1G.1         CTAGACCAACCTAAGATGTTTCCTCTAAAAATATTTCAGAAATTATTATACAA-TACTAG 1169
                          **    *  ***  *          **  **  * *** **   **** ** * **   *

9930_ACS1                TGGGTAAATGTATCATGAACAAAAA------TAGTAGT---CTATGATAGTTTACTATAG 1087
Gy14_ACS1                TGTATAAATGTATCATGAACAAAAA------TAGCAGT---CTATGATAGTTTACTATAG 1095
Gy14_ACS1G               TTGCTAAATAGATCCATTTTACAAATAATACGAGTAAGCTTCACAAATGGT-T-TTATTA 1089
DQ839406_ACS1G.1         TTGCTAAATAGATCCATTTTACAAATAATACGAGTAAGCTTCACAAATGGTTT-TTATTA 1228
                         *   *****  ***       * ***       ** *     *    ** ** *  ***

9930_ACS1                ATTGTGATATTTTAGTATAGTGATAAATGTT----TGTATAAATTTTATCATTTAAAATA 1143
Gy14_ACS1                ATTGTGATATTTTAGTATAGTGATAAATGTT----TGTATAAATTTTATCATTTAAAATA 1151
Gy14_ACS1G               CATGCATTATATGGACATTTTAAGTGATCTAATAAGACAATTATTTGACCCTATTACGAA 1149
DQ839406_ACS1G.1         CATGCATTATATGGACATTTTAAGTGATCTAATAAGACAATTATTTGACCCTATTACGAA 1288
                           **   *** *    **  * *   ** *        *   **** * * * * *   *
                                                             CArG transcription regulator
9930_ACS1                AAAATTGAGAGTAAAAATACATTTAAAAGAAAAAAGAAAAGAATGAATTAATTAGGAGG- 1202
Gy14_ACS1                AAATTCGAGAGTAAAAATACATTAAAAGAA-AAAAGAAAAGAATGAATTAATTAGGAGG- 1209
Gy14_ACS1G               AAAGACAATGTCAAAGAT----ATCTAGGCTGCCACTGGAGCATGCCCTCACTGAACAAA 1205
DQ839406_ACS1G.1         AAAGACAATGTCAAAGAT----ATCTAGGCTGCCACTGGAGCATGCCCTCACTGAACAAA 1344
                         ***    *    *** **        *       *    ** ***   * * *
```

```
9930_ACS1                ---AGAAAGAGTAATGGTAATGGATGTTAATTAAGGTGAAAGAAGAAAATGGCAGACAGA 1259
Gy14_ACS1                ---AG------AAAGAGTAATGGATGTTAATTAAGGTGAAAGAAGAAAATGGCAGACAGA 1260
Gy14_ACS1G               ATCAAACTGGCCTATTTTCCTTTCTTTTTT-TGTTTTTCATTAAAATAATC--ACTCCTT 1262
DQ839406_ACS1G.1         ATCAAACTGGCCTATTTTCCTTTCTTTTTT-TGTTTTTCATTAAAATAATC--ACTCCTT 1401
                            *         *   *  *   * **   *    *  *  ** * ***   *  *
                             -600                                           -550
9930_ACS1                GAGTGGCTGGTGATTGTGT-AATGAGGGAACACATTGAGCCCACCCCCACAGTTCATATT 1318
Gy14_ACS1                GAGTGGCTGGTGATTGTGT-AATGAGGGAACACATTGAGCCCACCCCCACAGTTCATATT 1319
Gy14_ACS1G               CATTGATATGCAACTTAGCAAAATATATAAATACTAGATAAGAAAACCC-AAATCAAGAG 1321
DQ839406_ACS1G.1         CATTGATATGCAACTTAGCAAAATATATAAATACTAGATAAGAAAACCC-AAATCAAGAG 1460
                          * **    *  * *  *  **  *   **    * **    *   **  *  ***

9930_ACS1                TTGATTTTTGAGGATCAGGTGGGTCTTTTTTCCCATCTTCTCTAACCCCTCCTGTCCCTC 1378
Gy14_ACS1                TTGATTTTTGAGGATCAGGTGGGTCTTTTTTCCCATCTTCTCTAACCCCTCCTGTCCCTC 1379
Gy14_ACS1G               AAGCAACGACAAAAACAGGGTGGTTT---AAGTATTTAACTACTTCGGACGGGCATAGTT 1378
DQ839406_ACS1G.1         AAGCAACGACAAAAACAGGGTGGTTT---AAGTATTTAACTACTTCGGACGGGCATAGTT 1517
                           *       *  * ****  *** *         *   **    *            *
                                            Distal promoter (start of BCAT 8th intron)
9930_ACS1                TATTTTCTTTCTTTCTATGTTTTCCCCTAACAAATTAACCCCCAAATCATTAACATTTTT 1438
Gy14_ACS1                TATTTTCTTTCTTTCTATGTTTTCCCCTAACAAATTAACCCCCAAATCATTAACATTTTT 1439
Gy14_ACS1G               ATGAGCAACATTAATTATGTTTTCCCCTAACAAATTAACCCCCAAATCATTAACATTTTT 1438
DQ839406_ACS1G.1         ATGAGCAACATTAATTATGTTTTCCCCTAACAAATTAACCCCCAAATCATTAACATTTTT 1577
                                    *   *********************************************
                                        -410 Proximal promoter start/braking point
9930_ACS1                CATTATCCCATTCATTTTTGTTTCATAATCCCCATTTTCATCTTCCATATCAAACCATAC 1498
Gy14_ACS1                CATTATCCCATTCATTTTTGTTTCATAATCCCCATTTTCATCTTCCATATCAAACCATAC 1499
Gy14_ACS1G               CATTATCCCATTCATTTTTGTTTCATAATCCCCATTTTCATCTTCCATATCAAACCATAC 1498
DQ839406_ACS1G.1         CATTATCCCATTCATTTTTGTTTCATAATCCCCATTTTCATCTTCCATATCAAACCATAC 1637
                         ************************************************************

9930_ACS1                CTGCTCTGGTCGGAGACACTTTCCATAAATAATTCCTCAAATCATCACTGTTTATCAACC 1558
Gy14_ACS1                CTGCTCTGGTCGGAGACACTTTCCATAAATAATTCCTCAAATCATCACTGTTTATCAACC 1559
Gy14_ACS1G               CTGCTCTGGTCGGAGACACTTTCCATAAATAATTCCTCAAATCATCACTGTTTATCAACC 1558
DQ839406_ACS1G.1         CTGCTCTGGTCGGAGACACTTTCCATAAATAATTCCTCAAATCATCACTGTTTATCAACC 1697
                         ************************************************************

9930_ACS1                CCCTTAATTAATTAATTAATATCAAACACACTCTCTATATTATCTCTACAATCTAAATAA 1618
Gy14_ACS1                CCCTTAATTAATTAATTAATATCAAACACACTCTCTATATTATCTCTACAATCTAAATAA 1619
Gy14_ACS1G               CCCTTAATTAATTAATTAATATCAAACACACTCTCTATATTATCTCTACAATCTAAATAA 1618
DQ839406_ACS1G.1         CCCTTAATTAATTAATTAATATCAAACACACTCTCTATATTATCTCTACAATCTAAATAA 1757
                         ************************************************************

9930_ACS1                TATACCTATATATATATATATTTGTCTCTTATTTTAATTAGACCAATTAAATTAATCTAT 1678
Gy14_ACS1                TATACCTATATATATATATATTTGTCTCTTATTTTAATTAGACCAATTAAATTAATCTAT 1679
Gy14_ACS1G               TATACCTATATATATATATATTTGTCTCTTATTTTAATTAGACCAATTAAATTAATCTAT 1678
DQ839406_ACS1G.1         TATACCTATATATATATATATTTGTCTCTTATTTTAATTAGACCAATTAAATTAATCTAT 1817
                         ************************************************************
                                               Auxin Responsive factor binding
9930_ACS1                AACCAAATCCCAGACCCCACATGTGGTCAAAAGTTTTTGTTGTCACCATCTACGTGGCCT 1738
Gy14_ACS1                AACCAAATCCCAGACCCCACATGTGGTCAAAAGTTTTTGTTGTCACCATCTACGTGGCCT 1739
Gy14_ACS1G               AACCAAATCCCAGACCCCACATGTGGTCAAAAGTTTTTGTTGTCACCATCTACGTGGCCT 1738
DQ839406_ACS1G.1         AACCAAATCCCAGACCCCACATGTGGTCAAAAGTTTTTGTTGTCACCATCTACGTGGCCT 1877
                         ************************************************************
                                                                      ABA responsive
9930_ACS1                ATGGATTTTGTCCTATAAATACCACCCTATGTTTGCTGAACACCTCTCCAACTCAAACAT 1798
Gy14_ACS1                ATGGATTTTGTCCTATAAATACCACCCTATGTTTGCTGAACACCTCTCCAACTCAAACAT 1799
Gy14_ACS1G               ATGGATTTTGTCCTATAAATACCACCCTATGTTTGCTGAACACCTCTCCAACTCAAACAT 1798
DQ839406_ACS1G.1         ATGGATTTTGTCCTATAAATACCACCCTATGTTTGCTGAACACCTCTCCAACTCAAACAT 1937
                         ************************************************************
                                      TATA box
9930_ACS1                TCGAAGAACTATCTACCATATTCCAACCAATACAAACCACCTCTTTTCTCTCTCTCTATT 1858
Gy14_ACS1                TCGAAGAACTATCTACCATATTCCAACCAATACAAACCACCTCTTTTCTCTCTCTCTATT 1859
Gy14_ACS1G               TCGAAGAACTATCTACCATATTCCAACCAATACAAACCACCTCTTTTCTCTCTCTCTATT 1858
DQ839406_ACS1G.1         TCGAAGAACTATCTACCATATTCCAACCAATACAAACCACCTCTTTTCTCTCTCTCTATT 1997
                         ************************************************************
                              ACS1 transcription start/5'UTR (+1, 197 bp from ATG)
9930_ACS1                TCTATTTCTCAACATTTCTCTCAATCTCATAATCTTTTGTACCTATATACCTCACCTCAA 1918
Gy14_ACS1                TCTATTTCTCAACATTTCTCTCAATCTCATAATCTTTTGTACCTATATACCTCACCTCAA 1919
Gy14_ACS1G               TCTATTTCTCAACATTTCTCTCAATCTCATAATCTTTTGTACCTATATACCTCACCTCAA 1918
DQ839406_ACS1G.1         TCTATTTCTCAACATTTCTCTCAATCTCATAATCTTTTGTACCTATATACCTCACCTCAA 2057
                         ************************************************************
```

```
9930_ACS1            CATTAATCTTAATCTTAAAATCATCAGATTCTTCTTCCATCCATCCCTAGGCTAGCTTAT 1978
Gy14_ACS1            CATTAATCTTAATCTTAAAATCATCAGATTCTTCTTCCATCCATCCCTAGGCTAGCTTAT 1979
Gy14_ACS1G           CATTAATCTTAATCTTAAAATCATCAGATTCTTCTTCCATCCATCCCTAGGCTAGCTTAT 1978
DQ839406_ACS1G.1     CATTAATCTTAATCTTAAAATCATCAGATTCTTCTTCCATCCATCCCTAGGCTAGCTTAT 2117
                     ************************************************************

9930_ACS1            TAGCAGCACAACCGAAGAAAAAATGAAGATGCTTTCCACAAAAGCCACGTGCAATTCCCA 2038
Gy14_ACS1            TAGCAGCACAACCGAAGAAAAAATGAAGATGCTTTCCACAAAAGCCACGTGCAATTCCCA 2038
Gy14_ACS1G           TAGCAGCACAACCGAAGAAAAAATGAAGATGCTTTCCACAAAAGCCACGTGCAATTCCCA 2038
DQ839406_ACS1G.1     TAGCAGCACAACCGAAGAAAAAATGAAGATGCTTTCCACAAAAGCCACGTGCAATTCCCA 2177
                     ************************************************************
                                           ACS1 CDS start (Exon 1, 147 bp)
9930_ACS1            CGGTCAAGATTCCTCCTACTTCTTAGGATGGGAAGCTTATGAGAAAAACCCCTTTGATGA 2098
Gy14_ACS1            CGGTCAAGATTCCTCCTACTTCTTAGGATGGGAAGCTTATGAGAAAAACCCCTTTGATGA 2098
Gy14_ACS1G           CGGTCAAGATTCCTCCTACTTCTTAGGATGGGAAGCTTATGAGAAAAACCCCTTTGATGA 2098
DQ839406_ACS1G.1     CGGTCAAGATTCCTCCTACTTCTTAGGATGGGAAGCTTATGAGAAAAACCCCTTTGATGA 2237
                     ************************************************************

9930_ACS1            GACTTCTAATCCCAACGGCATCATTCAGATGGGTCTTGCCGAGAATCAAGTAATTATATA 2158
Gy14_ACS1            GACTTCTAATCCCAACGGCATCATTCAGATGGGTCTTGCCGAGAATCAAGTAATTATATA 2158
Gy14_ACS1G           GACTTCTAATCCCAACGGCATCATTCAGATGGGTCTTGCCGAGAATCAAGTAATTATATA 2158
DQ839406_ACS1G.1     GACTTCTAATCCCAACGGCATCATTCAGATGGGTCTTGCCGAGAATCAAGTAATTATATA 2297
                     ************************************************************

9930_ACS1            TATTCTTCTTCGTATATATATATGTATACAAAAAAAATCACCATTTTTATGTTCTATTAA 2218
Gy14_ACS1            TATTCTTCTTCGTATATATATATGTATACAAAAAAAATCACCATTTTTATGTTCTATTAA 2218
Gy14_ACS1G           TATTCTTCTTCGTATATATATATGTATACAAAAAAAATCACCATTTTTATGTTCTATTAA 2218
DQ839406_ACS1G.1     TATTCTTCTTCGTATATATATATGTATACAAAAAAAATCACCATTTTTATGTTCTATTAA 2357
                     ************************************************************

9930_ACS1            GAGCTCTATTACTCACCTTTTTTTTTTTCACTTTTTTTTCAGCTATCATTTGATCTTCTT 2278
Gy14_ACS1            GAGCTCTATTACTCACCTTTTTTTTTTTCACTTTTTTTTCAGCTATCATTTGATCTTCTT 2278
Gy14_ACS1G           GAGCTCTATTACTCACCTTTTTTTTTTTCACTTTTTTTTCAGCTATCATTTGATCTTCTT 2278
DQ839406_ACS1G.1     GAGCTCTATTACTCACCTTTTTTTTTTTCACTTTTTTTTCAGCTATCATTTGATCTTCTT 2417
                     ************************************************************
                                                                ACS1 Exon 2 (291 bp)
9930_ACS1            GAATCATGGCTTACAAAAAATCCAGACGCAGCCAGCTTTAAACGTGATGGCAAATCAATT 2338
Gy14_ACS1            GAATCATGGCTTACAAAAAATCCAGACGCAGCCAGCTTTAAACGTGATGGCAAATCAATT 2338
Gy14_ACS1G           GAATCATGGCTTACAAAAAATCCAGACGCAGCCAGCTTTAAACGTGATGGCAA-TCAATT 2337
DQ839406_ACS1G.1     GAATCATGGCTTACAAAAAATCCAGACGCAGCCAGCTTTAAACGTGATGGCAAATCAATT 2477
                     ***************************************************** ******

9930_ACS1            TTTAGAGAGCTCGCTCTCTTCCAAGATTACCACGGCTTACCCGCATTCAAAAAGGCATTG 2398
Gy14_ACS1            TTTAGAGAGCTCGCTCTCTTCCAAGATTACCACGGCTTACCCGCATTCAAAAAGGCATTG 2398
Gy14_ACS1G           TTTAGAGAGCTCGCTCTCTTCCAAGATTACCACGGCTTACCCGCATTCAAAAAGGCATTG 2397
DQ839406_ACS1G.1     TTTAGAGAGCTCGCTCTCTTCCAAGATTACCACGGCTTACCCGCATTCAAAAAGGCATTG 2537
                     ************************************************************

9930_ACS1            GTAGAATTTATGGCGGAAATTAGAGGAAACAAAGTAACATTTGAAGCAAATAACATAGTC 2458
Gy14_ACS1            GTAGAATTTATGGCGGAAATTAGAGGAAACAAAGTAACATTTGAAGCAAATAACATAGTC 2458
Gy14_ACS1G           GTAGAATTTATGGCGGAAATTAGAGGAAACAAAGTAACATTTGAAGCAAATAACATAGTC 2457
DQ839406_ACS1G.1     GTAGAATTTATGGCGGAAATTAGAGGAAACAAAGTAACATTTGAAGCAAATAACATAGTC 2597
                     ************************************************************

9930_ACS1            CTTACAGCTGGCGCTACATCAGCCAATGAAACACTTATGTTCTGCCTTGCAGAGGCTGGC 2518
Gy14_ACS1            CTTACAGCTGGCGCTACATCAGCCAATGAAACACTTATGTTCTGCCTTGCAGAGGCTGGC 2518
Gy14_ACS1G           CTTACAGCTGGCGCTACATCAGCCAATGAAACACTTATGTTCTGCCTTGCAGAGGCTGGC 2517
DQ839406_ACS1G.1     CTTACAGCTGGCGCTACATCAGCCAATGAAACACTTATGTTCTGCCTTGCAGAGGCTGGC 2657
                     ************************************************************

9930_ACS1            GATGCCTTTCTCCTCCCAACTCCATACTATCCAGGGTTAGTAGTAATGATATTAATATTA 2578
Gy14_ACS1            GATGCCTTTCTCCTCCCAACTCCATACTATCCAGGGTTAGTAGTAATGATATTAATATTA 2578
Gy14_ACS1G           GATGCCTTTCTCCTCCCAACTCCATACTATCCAGGGTTAGTAGTAATGATATTAATATTA 2577
DQ839406_ACS1G.1     GATGCCTTTCTCCTCCCAACTCCATACTATCCAGGGTTAGTAGTAATGATATTAATATTA 2717
                     ************************************************************

9930_ACS1            ATTACTTTACATTCACTTAAACTTAAACATTTCCCTTTACTATAACTTCAAGCTAAATAA 2638
Gy14_ACS1            ATTACTTTACATTCACTTAAACTTAAACATTTCCCTTTACTATAACTTCAAGCTAAATAA 2638
Gy14_ACS1G           ATTACTTTACATTCACTTAAACTTAAACATTTCCCTTTACTATAACTTCAAGCTAAATAA 2637
DQ839406_ACS1G.1     ATTACTTTACATTCACTTAAACTTAAACATTTCCCTTTACTATAACTTCAAGCTAAATAA 2777
                     ************************************************************
```

```
9930_ACS1                 TTCTAATTAATCATGTATACTTTACTATAAACAGATTTGATAGAGATTTGAAATGGAGAA 2698
Gy14_ACS1                 TTCTAATTAATCATGTATACTTTACTATAAACAGATTTGATAGAGATTTGAAATGGAGAA 2698
Gy14_ACS1G                TTCTAATTAATCATGTATACTTTACTATAAACAGATTTGATAGAGATTTGAAATGGAGAA 2697
DQ839406_ACS1G.1          TTCTAATTAATCATGTATACTTTACTATAAACAGATTTGATAGAGATTTGAAATGGAGAA 2837
                          ************************************************************
                                                             ACS1 Exon 3 (1005 bp)
9930_ACS1                 CCGGAGTTGAGATTGTGCCAATTCATTGCACTAGCTCCAACGGCTTCCAAGTCACCCAAC 2758
Gy14_ACS1                 CCGGAGTTGAGATTGTGCCAATTCATTGCACTAGCTCCAACGGCTTCCAAGTCACCCAAC 2758
Gy14_ACS1G                CCGGAGTTGAGATTGTGCCAATTCATTGCACTAGCTCCAACGGCTTCCAAGTCACCCAAC 2757
DQ839406_ACS1G.1          CCGGAGTTGAGATTGTGCCAATTCATTGCACTAGCTCCAACGGCTTCCAAGTCACCCAAC 2897
                          ************************************************************

9930_ACS1                 CAGCTTTAGAACAAGCCTATCAAGAAGCCCAAGCTCGCAACCTACGTGTCAAAGGCGTAT 2818
Gy14_ACS1                 CAGCTTTAGAACAAGCCTATCAAGAAGCCCAAGCTCGCAACCTACGTGTCAAAGGCGTAT 2818
Gy14_ACS1G                CAGCTTTAGAACAAGCCTATCAAGAAGCCCAAGCTCGCAACCTACGTGTCAAAGGCGTAT 2817
DQ839406_ACS1G.1          CAGCTTTAGAACAAGCCTATCAAGAAGCCCAAGCTCGCAACCTACGTGTCAAAGGCGTAT 2957
                          ************************************************************

9930_ACS1                 TGGTTACCAACCCATCTAACCCATTGGGAACCACAATGACAAGAAATGAACTCGACTTGG 2878
Gy14_ACS1                 TGGTTACCAACCCATCTAACCCATTGGGAACCACAATGACAAGAAATGAACTCGACTTGG 2878
Gy14_ACS1G                TGGTTACCAACCCATCTAACCCATTGGGAACCACAATGACAAGAAATGAACTCGACTTGG 2876
DQ839406_ACS1G.1          TGGTTACCAACCCATCTAACCCATTGGGAACCACAATGACAAGAAATGAACTCGACTTGG 3017
                          ************************************************************

9930_ACS1                 TTTTTGATTTCATAACCTCCAAAGGCATTCATTTGATCAGCGACGAGATTTACTCTGGGA 2938
Gy14_ACS1                 TTTTTGATTTCATAACCTCCAAAGGCATTCATTTGATCAGCGACGAGATTTACTCTGGGA 2938
Gy14_ACS1G                TTTTTGATTTCATAACCTCCAAAGGCATTCATTTGATCAGCGACGAGATTTACTCTGGGA 2936
DQ839406_ACS1G.1          TTTTTGATTTCATAACCTCCAAAGGCATTCATTTGATCAGCGACGAGATTTACTCTGGGA 3077
                          ************************************************************

9930_ACS1                 CCGTTTTTGGGTCTCCAGGATTCGTGAGCGCCATGGAGGTGCTTAAGGAGAGGAGTAACG 2998
Gy14_ACS1                 CCGTTTTTGGGTCTCCAGGATTCGTGAGCGCCATGGAGGTGCTTAAGGAGAGGAGTAACG 2998
Gy14_ACS1G                CCGTTTTTGGGTCTCCAGGATTCGTGAGCGCCATGGAGGTGCTTAAGGAGAGGAGTAACG 2996
DQ839406_ACS1G.1          CCGTTTTTGGGTCTCCAGGATTCGTGAGCGCCATGGAGGTGCTTAAGGAGAGGAGTAACG 3137
                          ************************************************************

9930_ACS1                 AAGACGAGGAAGTTTGGAAGAGAGTTCATATTGTTTACAGTTTATCAAAGGATTTAGGGC 3058
Gy14_ACS1                 AAGACGAGGAAGTTTGGAAGAGAGTTCATATTGTTTACAGTTTATCAAAGGATTTAGGGC 3058
Gy14_ACS1G                AAGACGAGGAAGTTTGGAAGAGAGTTCATATTGTTTACAGTTTATCAAAGGATTTAGGGC 3056
DQ839406_ACS1G.1          AAGACGAGGAAGTTTGGAAGAGAGTTCATATTGTTTACAGTTTATCAAAGGATTTAGGGC 3197
                          ************************************************************

9930_ACS1                 TCCCAGGTTTTCGAGTTGGTGCAATTTACTCTAATGATGAAATGGTCGTGGCGGCTGCTA 3118
Gy14_ACS1                 TCCCAGGTTTTCGAGTTGGTGCAATTTACTCTAATGATGAAATGGTCGTGGCGGCTGCTA 3118
Gy14_ACS1G                TCCCAGGTTTTCGAGTTGGTGCAATTTACTCTAATGATGAAATGGTCGTGGCGGCTGCTA 3116
DQ839406_ACS1G.1          TCCCAGGTTTTCGAGTTGGTGCAATTTACTCTAATGATGAAATGGTCGTGGCGGCTGCTA 3257
                          ************************************************************

9930_ACS1                 CTAAAATGTCTAGCTTTGGGTTGGTGTCATCTCAAACACAATATCTTCTTTCAGCTATGC 3178
Gy14_ACS1                 CTAAAATGTCTAGCTTTGGGTTGGTGTCATCTCAAACACAATATCTTCTTTCAGCTATGC 3178
Gy14_ACS1G                CTAAAATGTCTAGCTTTGGGTTGGTGTCATCTCAAACACAATATCTTCTTTCAGCTATGC 3176
DQ839406_ACS1G.1          CTAAAATGTCTAGCTTTGGGTTGGTGTCATCTCAAACACAATATCTTCTTTCAGCTATGC 3317
                          ************************************************************

9930_ACS1                 TATCCGACAAGAAATTTACGAGAACCTATATTTCTGAGAATCAAAAGAGGTTGAAACAAA 3238
Gy14_ACS1                 TATCCGACAAGAAATTTACGAGAACCTATATTTCTGAGAATCAAAAGAGGTTGAAACAAA 3238
Gy14_ACS1G                TATCCGACAAGAAATTTACGAGAACCTATATTTCTGAGAATCAAAAGAGGTTGAAAC-AA 3235
DQ839406_ACS1G.1          TATCCGACAAGAAATTTACGAGAACCTATATTTCTGAGAATCAAAAGAGGTTGAAACAAA 3377
                          ********************************************************* **

9930_ACS1                 GACAGAAAATGTTGGTGAGTGGATTAGAGAAGGCTGGGATTAAGTGTTTGGAGAGTAATG 3298
Gy14_ACS1                 GACAGAAAATGTTGGTGAGTGGATTAGAGAAGGCTGGGATTAAGTGTTTGGAGAGTAATG 3298
Gy14_ACS1G                GACAGAAAATGTTGGTGAGTGGATTAGAGAAGGCTGGGATTAAGTGTTTGGAGAGTAATG 3295
DQ839406_ACS1G.1          GACAGAAAATGTTGGTGAGTGGATTAGAGAAGGCTGGGATTAAGTGTTTGGAGAGTAATG 3437
                          ************************************************************

9930_ACS1                 CTGGGTTGTTTTGTTGGGTTGATATGAGGCATTTGTTGGAATCAGATACGTTTGAATGTG 3358
Gy14_ACS1                 CTGGGTTGTTTTGTTGGGTTGATATGAGGCATTTGTTGGAATCAGATACGTTTGAATGTG 3358
Gy14_ACS1G                CTGGGTTGTTTTGTTGGGTTGATATGAGGCATTTGTTGGAATCAGATACGTTTGAATGTG 3354
DQ839406_ACS1G.1          CTGGGTTGTTTTGTTGGGTTGATATGAGGCATTTGTTGGAATCAGATACGTTTGAATGTG 3497
                          ************************************************************
```

```
9930_ACS1            AATTAAAGCTATGGAAGAAGATTGTTTACGAAGTGGGTTTGAATATTTCACCAGGATCAT 3418
Gy14_ACS1            AATTAAAGCTATGGAAGAAGATTGTTTACGAAGTGGGTTTGAATATTTCACCAGGATCAT 3418
Gy14_ACS1G           AATTAAAGCTATGGAAGAAGATTGTTTACGAAGTGGGTTTGAATATTTCACCAGGATCAT 3413
DQ839406_ACS1G.1     AATTAAAGCTATGGAAGAAGATTGTTTACGAAGTGGGTTTGAATATTTCACCAGGATCAT 3557
                     ************************************************************

9930_ACS1            CTTGTCATTGCACTGAACCAGGCTGGTTTCGTGTTTGTTTTGCTAATATGTCACAATCAA 3478
Gy14_ACS1            CTTGTCATTGCACTGAACCAGGCTGGTTTCGTGTTTGTTTTGCTAATATGTCACAATCAA 3478
Gy14_ACS1G           CTTGTCATTGCACTGAACCAGGCTGGTTTCGTGTTTGTTTTGCTAATATGTCACAATCAA 3473
DQ839406_ACS1G.1     CTTGTCATTGCACTGAACCAGGCTGGTTTCGTGTTTGTTTTGCTAATATGTCACAATCAA 3617
                     ************************************************************

9930_ACS1            CCTTGAAACTCGCTATCCGAAGATTGAAGTCGTTTGTTCAAGAATTGAGATCCGTTTCTA 3538
Gy14_ACS1            CCTTGAAACTCGCTATCCGAAGATTGAAGTCGTTTGTTCAAGAATTGAGATCCGTTTCTA 3538
Gy14_ACS1G           CCTTGAAACTCGCTATCCGAAGATTGAAGTCGTTTGTTCAAGAATTGAGATCCGTTTCTA 3533
DQ839406_ACS1G.1     CCTTGAAACTCGCTATCCGAAGATTGAAGTCGTTTGTTCAAGAATTGAGATCCGTTTCTA 3677
                     ************************************************************

9930_ACS1            CACCGAACGTTTCCACTACCACTACAAATATTCATGACAGCAAATTTTCCAAGAATATCA 3598
Gy14_ACS1            CACCGAACGTTTCCACTACCACTACAAATATTCATGACAGCAAATTTTCCAAGAATATCA 3598
Gy14_ACS1G           CACCGAACGTTTCCACTACCACTACAAATATTCATGACAGCAAATTTTCCAAGAATATCA 3593
DQ839406_ACS1G.1     CACCGAACGTTTCCACTACCACTACAAATATTCATGACAGCAAATTTTCCAAGAATATCA 3737
                     ************************************************************

9930_ACS1            AGAAAAATATTTTCACCAAATGGGTTTTCCGGCAATCGGTTCAAGATCAAGCCAACCGCA 3658
Gy14_ACS1            AGAAAAATATTTTCACCAAATGGGTTTTCCGGCAATCGGTTCAAGATCAAGCCAACCGCA 3658
Gy14_ACS1G           AGAAAAATATTTTCACCAAATGGGTTTTCCGGCAATCGGTTCAAGATCAAGCCAACCGCA 3653
DQ839406_ACS1G.1     AGAAAAATATTTTCACCAAATGGGTTTTCCGGCAATCGGTTCAAGATCAAGCCAACCGCA 3797
                     ************************************************************

9930_ACS1            AGCTCCATTATGAACGATAGCCTGGTTTTTTT-TTTTTTTACTCATCATCCATATTTGGG 3717
Gy14_ACS1            AGCTCCATTATGAACGATAGCCTGGTTTTTTTTTTTTTTTACTCATCATCCATATTTGGG 3718
Gy14_ACS1G           AGCTCCATTATGAACGATAGCCTGGTTTTTTTTTTTTTTTACTCATCATCCATATTTGGG 3712
DQ839406_ACS1G.1     AGCTCCATTATGAACGATAGCCTGGTTTTTTTTTTTTTTTACTCATCATCCATATTTGGG 3857
                     ******************************** ***************************
                                       Translation stop codon
9930_ACS1            GGAATTTTTTTTTCTTAATCTTGTTGAAATATTCCAAATTTAGAATTTTCATATTGTAAT 3777
Gy14_ACS1            GGAATTTTTTTTTCTTAATCTTGTTGAAATATTCCAAATTTAGAATTTTCATATTGTAAT 3778
Gy14_ACS1G           GGAATTTTTTTTTCTTAATCTTGTTGAAATATTCCAAATTTAGAATTTTCATATTGTAAT 3772
DQ839406_ACS1G.1     GGAATTTTTTTTTCTTAATCTTGTTGAAATATTCCAAATTTAGAATTTTCATATTGTAAT 3917
                     ************************************************************

9930_ACS1            TAGAGAAAAAAAGAAAAAGAAAAGTGCACTCGGTCCATTGTGGGATTAGTAGTGGCTTTA 3837
Gy14_ACS1            TAGAGAAAAAAAGAAAAAGAAAAGTGCACTCGGTCCATTGTGGGATTAGTAGTGGCTTTA 3838
Gy14_ACS1G           TAGAGAAAAAAAGAAAAAGAAAAGTGCACTCGGTCCATTGTGGGATTAGTAGTGGCTTTA 3832
DQ839406_ACS1G.1     TAGAGAAAAAAAGAAAAAGAAAAGTGCACTCGGTCCATTGTGGGATTAGTAGTGGCTTTA 3977
                     ************************************************************

9930_ACS1            ATTAATTAAATTAATTAATTATTAATCTTTTTTTTCATTAATTCTGATTCTCTCTCTCTC 3897
Gy14_ACS1            ATTAATTAAATTAATTAATTATTAATCTTTTTTTTTCATTAATTCTGATTCTCTCTCTCTC 3898
Gy14_ACS1G           ATTAATTAAATTAATTAATTATTAATCTTTTTTTTCATTAATTCTGATTCTCTCTCTCTC 3891
DQ839406_ACS1G.1     ATTAATTAAATTAATTAATTATTAATCTTTTTTTTCATTAATTCTGATTCTCTCTCTCTC 4037
                     ************************************************************
                                              mRNA transcript end
9930_ACS1            TCTCTCTCTCTCTCTCTCTAACATCAGAATATTTAATTGAAGTATATTAGAATCGAATGA 3957
Gy14_ACS1            TCTCTCTCTCTCTCTCTCTAACATCAGAATATTTAATTGAAGTATATTAGAATCGAATGA 3958
Gy14_ACS1G           TCTCTCTCTCTCTCTCTCTAACATCAGAATATTTAATTGAAGTATATTAGAATCGAATGA 3951
DQ839406_ACS1G.1     TCTCTCTCTCTCTCTCTCTAACATCAGAATATTTAATTGAAGTATATTAGAATCGAATGA 4097
                     ************************************************************

9930_ACS1            ATTTTCCAACGTGGAGGATTTTATGCGAGATGTTATCATGTGTTGAAACGATAATGGTTG 4017
Gy14_ACS1            ATTTTCCAACGTGGAGGATTTTATGCGAGATGTTATCATGTGTTGAAACGATAATGGTTG 4018
Gy14_ACS1G           ATTTTCCAACGTGGAGGATTTTATGCGAGATGTTATCATGTGTTGAAACGATAATGGTTG 4011
DQ839406_ACS1G.1     ATTTTCCAACGTGGAGGATTTTATGCGAGATGTTATCATGTGTTGAAACGATAATGGTTG 4157
                     ************************************************************

9930_ACS1            GCCATGTGAGAAGATTTTATTTATTAATCAATTATTATTATTATTATTATTGTTTAATGT 4077
Gy14_ACS1            GCCATGTGAGAAGATTTTATTTATTAATCAATTATTATTATTATTATTATTGTTTAATGT 4078
Gy14_ACS1G           GCCATGTGAGAAGATTTTATTTATTAATCAATTATTATTATTATTATTATTGTTTAATGT 4071
DQ839406_ACS1G.1     GCCATGTGAGAAGATTTTATTTATTAATCAATTATTATTATTATTATTATTGTTTAATGT 4217
                     ************************************************************
```

```
9930_ACS1            ATAGAGAAAATAATAATAATAATAATATAGAAAGAGATGGCTTGCAAAGTGAAACGGCGC 4137
Gy14_ACS1            ATAGAGAAAATAATAATAATAATAATATAGAAAGAGATGGCTTGCAAAGTGAAACGGCGC 4138
Gy14_ACS1G           ATAGAGAAAATAATAATAATAATAATATAGAAAGAGATGGCTTGCAAAGTGAAACGGCGC 4131
DQ839406_ACS1G.1     ATAGAGAAAATAATAATAATAATAATATAGAAAGAGATGGCTTGCAAAGTGAAACGGCGC 4277
                     ************************************************************

9930_ACS1            CGTTGGCGAGAATAGCGGCTGAAAGACGGTGGGTTTTT-TTTTTAATAGTAAGAAGATGT 4196
Gy14_ACS1            CGTTGGCGAGAATAGCGGCTGAAAGACGGTGGGTTTTTTTTTTTAATAGTAAGAAGATGT 4198
Gy14_ACS1G           CGTTGGCGAGAATAGCGC-TGAAAGACGGTGGTTTTT-TTTTTTAATAGTAAGAAGATGT 4189
DQ839406_ACS1G.1     CGTTGGCGAGAATAGCGGCTGAAAGACGGTGGGTTTT-TTTTTTAATAGTAAGAAGATGT 4336
                     *****************  ************* ****  *********************

9930_ACS1            GCCGACATGAACCGGTTTAATTTGGTTGAGTCATTGGTTACCCAACCGAAACCGACCTCT 4256
Gy14_ACS1            GCCGACATGAACCGGTTTAATTTGGTTGAGTCATTGGTTACCCAACCGAAACCGACCTCT 4258
Gy14_ACS1G           GCCGACATGAACCGGTTTAATTTGGTTGAGTCATTGGTTACCCAACCGAAACCGACCTCT 4249
DQ839406_ACS1G.1     GCCGACATGAACCGGTTTAATTTGGTTGAGTCATTGGTTACCCAACCGAAACCGACCTCT 4396
                     ************************************************************

9930_ACS1            TTCTTTCACACAAAACCCAAATACACAAATAATAATAATAATAAGTGTGTGATTTTAAGC 4316
Gy14_ACS1            TTCTTTCACACAAAACCCAAATACACAAATAATAATAATAATAAGTGTGTGATTTTAAGC 4318
Gy14_ACS1G           TTCTTTCACACAAAACCCAAATACACAAATAATAATAAT-ATAAGTGTGTGATTTTAAGC 4308
DQ839406_ACS1G.1     TTCTTTCACACAAAACCCAAATACACAAATAATAATAATAATAAGTGTGTGATTTTAAGC 4456
                     *************************************** ********************

9930_ACS1            AATTTGTGTGTTGTGTTGTACAGCAAAAGAAGGCCAATCCCAAGTGGGTTGTTGGTCAGA 4376
Gy14_ACS1            AATTTGTGTGTTGTGTTGTACAGCAAAAGAAGGCCAATCCCAAGTGGGTTGTTGGTCAGA 4378
Gy14_ACS1G           AATTTGTGTGTTGTGTTGTACAGCAAAAGAAGGCCAATCCCAAGT-GGTTGTTGGTCAGA 4367
DQ839406_ACS1G.1     AATTTGTGTGTTGTGTTGTACAGCAAAAGAAGGCCAATCCCAAGTGGGTTGTTGGTCAGA 4516
                     ********************************************* **************

9930_ACS1            ATAGCCGAAGACAGTCAAACTATTACGTTTGCCTAGGCCCTCCAAGAATCAAACTTTGTC 4436
Gy14_ACS1            ATAGCCGAAGACAGTCAAACTATTACGTTTGCCTAGGCCCTCCAAGAATCAAACTTTGTC 4438
Gy14_ACS1G           ATAGCCGAAGACAGTCAAACTATTACGTTTGCCTAGGCCCTCCAAGAATCAAACTTTGTC 4427
DQ839406_ACS1G.1     ATAGCCGAAGACAGTCAAACTATTACGTTTGCCTAGGCCCTCCAAGAATCAAACTTTGTC 4576
                     ************************************************************

9930_ACS1            CCCATTTCAACATTTCAACATTTCTTTTTCTATTTTTTTTTTCCTTTTCGTTAACCTATA 4496
Gy14_ACS1            CCCATTTCAACATTTCAACATTTCTTTTTCTATTTTTTTTTTCCTTTTCGTTAACCTATA 4498
Gy14_ACS1G           CCCATTTCAACATTTCAACATTTCTTTTTCTATTTTT-TTTTCCTTTTCGTTAACCTATA 4486
DQ839406_ACS1G.1     CCCATTTCAACATTTCAACATTTCTTTTTCTATTTTTTTTTTCCTTTTCGTTAACCTATA 4636
                     ************************************* **********************

9930_ACS1            TATAAAATCTCACTCTTTTTTATTTTCTTTTAAACCATTTTAAATTAACTCTAATCAAAT 4556
Gy14_ACS1            TATAAAATCTCACTCTTTTTTATTTTCTTTTAAACCATTTTAAATTAACTCTAATCAAAT 4558
Gy14_ACS1G           TATAAAATCTCACTCTTTTTTATTTTCTTTTAAACCATTTTAAATTAACTCTAATCAAAT 4546
DQ839406_ACS1G.1     TATAAAATCTCACTCTTTTTTATTTTCTTTTAAACCATTTTAAATTAACTCTAATCAAAT 4696
                     ************************************************************

9930_ACS1            TATTGGATATTATTGATCTCACATGACGATGCTTCTTCAAATTGCTTTATAAAAAATTATG 4616
Gy14_ACS1            TATTGGATATTATTGATCTCACATGACGATGCTTCTTCAAATTGCTTTATAAAAAATTATG 4618
Gy14_ACS1G           TAT-TGATATTATTGATCTCACATGACGATGCTTCTTCAAATTGCTTTATAAAAAATTATG 4605
DQ839406_ACS1G.1     TATTGGATATTATTGATCTCACATGACGATGCTTCTTCAAATTGCTTTATAAAAAATTATG 4756
                     ***  *******************************************************

9930_ACS1            ACATTAATTATATATAGGATAAATCCAAAAAATAATTGAGTCAAAGTATTAAATTAAACT 4676
Gy14_ACS1            ACATTAATTATATATAGGATAAATCCAAAAAATAATTGAGTCAAAGTATTAAATTAAACT 4678
Gy14_ACS1G           ACATTAATTATATATAGGATAAATCCAAAAAATAATTGAGTCAAAGTATTAAATTAAACT 4665
DQ839406_ACS1G.1     ACATTAATTATATATAGGATAAATCCAAAAAATAATTGAGTCAAAGTATTAAATTAAACT 4816
                     ************************************************************

9930_ACS1            TT---------------      4678
Gy14_ACS1            -----------------      4678
Gy14_ACS1G           TTGTCTTT---------      4673
DQ839406_ACS1G.1     TTGTCTTTATTTTACTT      4833
```

Supplementary File 5 (Table S2). Sex expression of the cucumber materials used in this study.

#	**Accessions**	**Sex expression***	**Objectives**
1	WI2757	G	G01 (Fig. 3)
2	2A	G	G02 (Fig. 3)
3	NZ1	G	G03 (Fig. 3)
4	GP5818	G	G04 (Fig. 3)
5	Gy14	G	G05 (Fig. 3)
6	PI 308915	G	G06 (Fig. 3)
7	1351A1	G	G07 (Fig. 3)
8	7155B	G	G08 (Fig. 3)
9	BA-m10-139	G	G09 (Fig. 3)
10	ZS0806	G	G10 (Fig. 3)
11	ZS0833	G	G11 (Fig. 3)
12	DP1	G	G12 (Fig. 3)
13	MSU 713-5	G	G13 (Fig. 3)
14	AM297	G	G14 (Fig. 3)
15	PI 292012	H	H01 (Fig. 3)
16	PI 356809	H	H02 (Fig. 3)
17	PI 370643	H	H03 (Fig. 3)
18	PI 500365	H	H04 (Fig. 3)
19	WI1983H	H	H05 (Fig. 3)
20	WI7012A	M	M01 (Fig. 3)
21	Midget	M	M02 (Fig. 3)
22	PI 267942	M	M03 (Fig. 3)
23	PI 271327	M	M04 (Fig. 3)
24	PI 271328	M	M05 (Fig. 3)
25	SV4719S-M	M	M07 (Fig. 3)
26	Northern Pickling	M	M08 (Fig. 3)
27	Gy14*WI7284 F1	SubG	SubG01 (Fig. 3)
28	Gy14*AM056 F1	SubG	SubG02 (Fig. 3)
29	AM007 x AM001 F1	SubG	SubG03 (Fig. 3)
30	Gy14M-17	M	CNV UCO, qPCR
31	Gy14M-42	M	CNV UCO, qPCR
32	Gy14M-63	M	CNV UCO, qPCR
33	9930	M	Reference
34	PI 183967	M	CNV, reads alignment
35	PI 197088	M	CNV, reads alignment
36	WI7167	M	CNV, reads alignment
37	G421	G	CNV, reads alignment

* From 2014 and 2015 field data. A = andromonoecious; G = gynoecious, M = monoecious, H = hermaphrodite.

Supplementary File 6 (Table S3. Nucleotide diversity at the 50 kb *F* locus (*CsACS1G*) among 48 cucumber varieties.

Serial #	PI or CGN #	Accession names	Sex expression	Botanical groups	Origin of accessions	Haplotypes[a] 24,207,363	24,207,424	24,207,547	24,207,976	24,208,099	24,208,574	24,209,013	24,209,048	24,209,091	24,209,317	24,209,361	24,209,584	24,211,499	24,211,585	24,216,268	24,218,009	24,218,987	24,220,561	24,221,073	24,221,548
																			ACS1	ACS1	ACS1	ACS1			
1	CGN19579	1972B-2	G	*C.s.* var. *sativus*	United States	T	G	A	A	G	C	C	C	G	C	T	C	C	A	C	A	T	T	C	T
2	CGN19677	M5	G	*C.s.* var. *sativus*	Netherlands	T	G	A	A	G	C	C	C	G	C	T	C	C	A	C	A	T	T	C	T
3	CGN19844	2163	G	*C.s.* var. *sativus*	United States	T	G	A	A	G	C	C	C	G	C	T	C	C	A	C	A	T	T	C	T
4	CGN20515	Gy3(S4)	G	*C.s.* var. *sativus*	Poland	T	G	A	A	G	T	C	C	G	C	T	C	C	A	C	A	T	T	C	T
5	G421	G421	G	*C.s.* var. *sativus*	United States	T	G	A	A	G	T	C	C	G	C	T	C	C	A	C	A	T	T	C	A
6	G8	G8	G	*C.s.* var. *sativus*	United States	T	G	A	A	G	T	C	C	G	C	T	C	C	A	C	A	T	T	C	T
7	Gy14	Gy14	G	*C.s.* var. *sativus*	United States	T	G	A	A	G	T	C	C	G	C	T	C	C	A	C	A	T	T	C	T
8	NP1	NP1	G	*C.s.* var. *sativus*	United States	T	G	A	A	G	T	C	C	G	C	T	C	C	A	C	A	T	T	C	A
9	P1	P1	G	*C.s.* var. *sativus*	United States	T	G	A	A	G	T	C	C	G	C	T	C	C	A	C	A	T	T	C	A
10	WI2757	WI2757	G	*C.s.* var. *sativus*	United States	T	G	A	A	G	T	C	C	G	C	T	C	C	A	C	A	T	T	C	A
11	PI 221440	Khiar; Badreng-i-Savareh	M	*C.s.* var. *sativus*	Afghanistan	T	G	A	A	G	C	C	C	G	C	T	C	C	A	C	A	T	T	C	T
12	CGN20243	Cus124/71	M	*C.s.* var. *sativus*	China	T	G	A	A	G	T	C	C	G	C	T	C	C	A	C	A	T	T	C	T
13	V05A0522	Huang Gua	M	*C.s.* var. *sativus*	China	C	A	G	G	G	T	C	C	G	C	T	C	C	A	C	A	T	T	C	T
14	V05A0552	Qing Dao Qiu Ye Er San	M	*C.s.* var. *sativus*	China	T	G	A	A	G	T	C	C	G	C	T	C	C	A	C	G	T	T	C	T
15	V05A0985	Ye San Bai	M	*C.s.* var. *sativus*	China	T	G	G	G	G	T	C	C	A	C	T	C	C	A	C	A	T	T	C	A
16	V05A1115	Qian Qi Li Huang Gua	M	*C.s.* var. *sativus*	China	T	G	A	A	G	T	C	C	G	C	T	C	C	A	C	A	T	T	C	T
17	CG9142	Ban Na Huang Gua 12	M	*C.s.* var. *xishuangbannesis*	China, Yunnan	T	G	A	A	G	C	C	C	G	C	T	C	C	A	C	A	T	T	C	A
18	CG9153	Bai Chang Yuan Huang Gua	M	*C.s.* var. *xishuangbannesis*	China, Yunnan	T	G	A	A	G	C	C	C	G	C	T	C	C	A	C	A	T	T	C	T
19	CG9164	Yuan Bai Huang Gua	M	*C.s.* var. *xishuangbannesis*	China, Yunnan	C	G	G	G	G	C	C	C	G	C	T	C	C	A	C	A	T	T	C	T
20	CG9169	Yuan Duan Huang Pi	M	*C.s.* var. *xishuangbannesis*	China, Yunnan	T	G	A	A	G	C	C	C	G	C	T	C	C	A	C	A	T	T	C	T
21	CG9185	Ban Na Huang Gua 51	M	*C.s.* var. *xishuangbannesis*	China, Yunnan	T	G	A	A	G	C	C	C	G	C	T	C	C	A	C	A	T	T	C	T
22	PI 288995	Nemet Kigyo	M	*C.s.* var. *sativus*	Hungary	T	G	A	A	G	C	C	C	G	C	T	C	C	A	C	A	T	T	C	T
23	PI 326594	Kecskemeti Hamvas	M	*C.s.* var. *sativus*	Hungary	T	G	A	A	G	C	C	C	G	C	T	C	C	A	C	A	T	T	C	T
24	13598	13598	M	*C.s.* var. *hardwickii*	India	C	A	G	G	T	T	T	T	A	T	T	C	C	A	C	G	A	A	G	T
25	CUS 233	CUS 233	M	*C.s.* var. *hardwickii*	India	C	A	G	G	T	T	T	T	A	T	T	C	T	T	C	A	T	T	C	T
26	Hw 2	Hw 2	M	*C.s.* var. *hardwickii*	India	C	A	G	G	T	C	T	T	A	T	T	C	C	A	C	A	T	T	C	A
27	Hw 5	Hw 5	M	*C.s.* var. *hardwickii*	India	C	A	G	G	T	T	T	C	A	T	T	C	C	A	C	A	T	T	C	T
28	LJ90430	LJ 90430	M	*C.s.* var. *hardwickii*	India	C	A	G	G	T	T	T	C	G	T	T	C	C	T	C	A	T	T	C	T
29	PI 504564	VIR 3147	M	*C.s.* var. *hardwickii*	India	C	A	G	G	T	T	T	C	G	T	T	C	C	A	C	A	T	T	C	T
30	PI 605988	USM 411	M	*C.s.* var. *sativus*	India	C	A	G	G	T	C	T	T	G	T	A	C	T	A	C	A	T	T	C	A
31	PI 606033	Poona Kheera	M	*C.s.* var. *sativus*	India	C	A	G	G	T	T	T	T	G	T	T	C	C	A	C	A	T	T	C	A
32	VIR 3147	VIR 3147	M	*C.s.* var. *hardwickii*	India	C	A	G	G	T	T	T	C	G	T	T	C	C	A	C	A	T	T	C	T
33	CG3010	Sagami Hanpaku Fushinari Kyuri	M	*C.s.* var. *sativus*	Japan	C	A	G	G	T	T	T	T	A	T	A	T	T	T	G	G	A	T	G	A
34	CGN19828	CGN19828	M	*C.s.* var. *sativus*	Japan	C	A	G	G	T	T	T	T	A	T	A	T	T	T	G	G	A	T	G	T
35	CGN20854	Aonaga Suyo Kyuri	M	*C.s.* var. *sativus*	Japan	C	A	G	G	T	T	T	T	A	T	A	T	T	T	G	G	A	A	G	T
36	PI 267942	Sekino No. 2 (Ochiai No.2)	M	*C.s.* var. *sativus*	Japan	C	A	G	G	T	T	T	T	A	T	A	T	T	T	G	G	A	A	G	A
37	PI 504563	Tsuda	M	*C.s.* var. *sativus*	Japan	C	A	G	G	T	T	T	T	A	T	A	T	T	T	G	G	A	A	G	A
38	CGN20512	752	M	*C.s.* var. *sativus*	Netherlands	T	G	A	A	G	C	C	C	G	C	T	C	C	A	C	A	T	T	C	T
39	PI 263047	Altaisky Ranny	M	*C.s.* var. *sativus*	Russia	T	G	A	A	G	C	C	C	G	C	T	C	C	A	C	A	T	T	C	A
40	PI 512596	A-C-3	M	*C.s.* var. *sativus*	Spain	T	G	A	A	G	C	C	C	G	C	T	C	C	A	C	A	T	T	C	T
41	PI 338236	Ames 1208	M	*C.s.* var. *sativus*	Turkey	T	G	A	A	G	C	C	C	G	C	T	C	C	A	C	A	T	T	C	A
42	CGN19655	SC 53-B (6)	M	*C.s.* var. *sativus*	United States	T	G	A	A	G	C	C	C	G	C	T	C	C	A	C	A	T	T	C	T
43	CGN21626	National Pickle	M	*C.s.* var. *sativus*	United States	T	G	A	A	G	C	C	C	G	C	T	C	C	A	C	A	T	T	C	T
44	CGN21627	Spartan Garden	M	*C.s.* var. *sativus*	United States	T	G	A	A	G	C	C	C	G	C	T	C	C	A	C	A	T	T	C	T
45	PI 500359	ZM 1065	M	*C.s.* var. *sativus*	Zambia	C	A	G	G	T	T	T	T	A	T	T	C	C	A	C	A	T	T	G	A
46	PI 500361	Inyangu	M	*C.s.* var. *sativus*	Zambia	C	A	G	G	T	C	T	T	A	T	T	C	C	A	C	A	T	T	C	A
47	PI 500365	Chibimbi	M	*C.s.* var. *sativus*	Zambia	C	A	G	G	T	T	T	T	A	T	T	C	C	A	C	A	T	T	C	A
48	PI 482412	TGR 580	M	*C.s.* var. *sativus*	Zimbabwe	C	A	G	G	T	T	T	T	G	T	T	C	C	A	C	A	T	T	C	A

[a] The SNP positions are based on 9930 V2.0. Genome data are from Yang et al. (2012), and Qi et al. (2013). The repeat unit of the *F* locus (~30 kb) is from 24,217,232 to 24,247,474 in 9930 V2.0.

24,222,140	24,223,241	24,223,245	24,226,857	24,227,034	24,227,349	24,229,168	24,229,194	24,229,482	24,229,619	24,233,704	24,234,010	24,236,089	24,236,195	24,236,212	24,236,380	24,236,801	24,237,028	24,237,206	24,237,254	24,238,678	24,238,966	24,240,300	24,242,378	24,244,219	24,245,834	24,245,869	24,245,888	24,246,390	24,247,113
		MYB	MYB	MYB	MYB	MYB	MYB	MYB	MYB	MYB	MYB	MYB	MYB	MYB	MYB	MYB	MYB	MYB	MYB						BCAT	BCAT	BCAT	BCAT	BCAT
C	G	G	T	T	A	A	-	G	G	A	C	A	C	A	T	G	G	T	T	G	T	C	G	G	A	T	T	G	A
C	G	G	T	T	A	A	A	G	G	A	C	A	C	A	T	G	G	T	T	G	T	C	G	A	A	T	T	G	A
C	G	G	T	T	A	G	-	G	G	A	C	A	C	A	T	G	G	T	T	G	T	C	G	A	A	T	T	G	A
C	G	G	T	T	A	A	A	G	G	A	C	A	C	A	T	G	G	T	T	G	T	C	G	A	A	T	T	G	A
C	G	G	T	T	A	A	A	G	G	A	C	A	C	A	T	G	G	T	T	G	T	C	G	A	A	T	T	G	A
C	G	G	T	T	A	G	A	G	G	A	C	A	C	A	T	G	G	T	T	G	T	C	G	A	A	T	T	G	A
C	G	G	T	T	A	A	A	G	G	A	C	A	C	A	T	G	G	T	T	G	T	C	G	A	A	T	T	G	A
C	G	G	T	T	A	G	A	G	G	A	C	A	C	A	T	G	G	T	T	G	T	C	G	A	A	T	T	G	A
C	G	G	T	T	A	G	A	G	G	A	C	A	C	A	T	G	G	T	T	G	T	C	G	A	A	T	T	G	A
C	G	G	T	T	A	A	A	G	G	A	C	A	C	A	T	G	G	T	T	G	T	C	G	A	A	T	T	G	A
C	G	G	T	T	A	A	A	G	G	A	C	A	C	A	T	G	G	T	T	G	T	C	A	A	A	C	-	T	A
C	G	G	T	T	A	G	A	G	G	A	C	A	C	A	T	G	G	T	T	G	C	C	A	A	A	C	-	T	A
C	G	G	T	T	A	G	A	G	G	A	C	A	C	A	T	G	G	T	T	G	C	C	A	A	A	C	-	T	A
C	G	G	T	A	C	G	-	G	G	A	C	A	C	A	T	G	G	T	T	G	C	C	A	G	A	C	-	T	A
C	G	G	T	T	A	G	-	G	G	A	C	A	C	A	T	G	G	T	T	G	C	C	A	G	A	C	-	T	A
C	G	G	T	T	A	G	-	G	G	A	C	A	C	A	T	G	G	T	T	G	C	C	A	A	A	C	-	T	A
C	G	G	A	A	C	A	-	A	C	C	T	G	A	G	C	A	C	C	C	T	T	A	G	G	T	C	-	G	C
C	G	G	A	A	C	A	A	A	C	C	T	G	A	G	C	A	C	C	C	T	T	A	G	G	T	C	-	G	C
C	G	G	A	A	C	A	A	A	C	C	T	G	A	G	C	A	C	C	C	T	T	A	G	G	T	C	-	G	C
C	G	G	A	A	C	A	A	A	C	C	T	G	A	G	C	A	C	C	C	T	T	A	G	G	T	C	-	G	C
C	G	G	A	A	C	A	A	A	C	C	T	G	A	G	C	A	C	C	C	T	T	A	G	G	T	C	-	G	C
C	G	G	T	T	A	A	A	G	G	A	C	A	C	A	T	G	G	T	T	G	C	C	A	A	A	C	-	T	A
C	G	G	T	T	A	A	A	G	G	A	C	A	C	A	T	G	G	T	T	G	C	C	A	A	A	C	-	T	A
T	A	A	T	T	A	A	A	G	G	C	C	A	A	G	C	A	C	C	C	T	T	A	A	G	A	C	-	T	A
C	G	G	T	T	A	G	-	G	G	C	T	G	A	G	C	A	C	C	C	T	T	C	A	A	A	C	-	T	A
T	A	A	T	T	C	A	A	G	G	C	T	A	A	G	C	A	C	C	C	T	T	A	A	G	A	C	-	T	A
T	A	A	T	T	C	A	A	G	G	C	T	A	A	G	C	A	C	C	C	T	T	A	A	A	A	C	-	T	A
C	A	G	T	T	C	G	-	G	C	C	T	A	A	G	C	A	C	C	C	T	T	A	A	G	A	C	-	T	A
C	G	G	T	A	C	A	A	G	G	C	T	G	A	G	C	A	C	C	C	T	C	A	A	A	A	C	-	T	A
C	G	G	T	T	A	A	A	G	G	A	C	A	C	A	T	G	G	T	T	G	C	C	A	A	A	C	-	T	A
C	G	G	T	T	A	A	A	G	G	A	C	A	C	A	T	G	G	T	T	G	C	C	A	A	A	C	-	T	A
C	G	G	T	T	C	A	A	G	C	C	C	G	A	G	C	A	C	C	C	T	C	C	A	A	A	C	-	T	A
T	A	A	A	A	C	A	-	A	C	C	T	G	A	G	C	A	C	C	C	T	T	A	G	G	T	C	-	G	C
T	G	G	A	A	C	A	-	A	C	C	T	G	A	G	C	A	C	C	C	T	T	A	G	G	T	C	-	G	C
T	A	A	A	A	C	A	-	A	C	C	T	G	A	G	C	A	C	C	C	T	T	A	G	G	T	C	-	T	C
T	A	A	A	A	C	G	-	A	C	C	T	G	A	G	C	A	C	C	C	T	T	A	G	G	T	C	-	G	C
T	G	G	A	A	C	G	-	A	C	C	T	G	A	G	C	A	C	C	C	T	T	A	G	G	T	C	-	G	C
C	G	G	T	T	A	A	-	G	G	A	C	A	C	A	T	G	G	T	T	G	C	C	A	A	A	C	-	T	A
C	G	G	T	T	A	A	A	G	G	A	C	A	C	A	T	G	G	T	T	G	C	C	A	G	A	T	-	T	A
C	G	G	T	T	A	A	A	G	G	A	C	A	C	A	T	G	G	T	T	G	C	C	A	A	A	C	-	T	A
C	G	G	T	T	A	A	A	G	G	A	C	A	C	A	T	G	G	T	T	G	C	C	A	G	A	T	-	T	A
C	G	G	T	T	A	A	A	G	G	A	C	A	C	A	T	G	G	T	T	G	C	A	A	A	A	C	-	T	A
C	G	G	T	T	A	A	-	G	G	A	C	A	C	A	T	G	G	T	T	G	T	C	A	G	A	C	-	T	A
C	G	G	T	T	A	A	A	G	G	A	C	A	C	A	T	G	G	T	T	G	C	C	A	A	A	C	-	T	A
C	A	G	T	A	C	A	A	G	G	A	C	A	C	A	T	G	G	T	T	G	C	C	A	A	A	C	-	T	A
C	G	G	T	A	C	A	A	G	G	A	C	A	C	A	T	G	G	T	T	G	C	C	A	A	A	C	-	T	A
C	A	G	T	A	C	A	A	G	G	A	C	A	C	A	T	G	G	T	T	G	C	C	A	A	A	C	-	T	A
C	G	G	T	T	A	A	A	G	G	A	C	A	C	A	T	G	G	T	T	G	C	C	A	A	A	C	-	T	A

24,247,129	24,247,448	24,247,478	24,247,501	24,247,824	24,247,936	24,248,880	24,249,294	24,249,438	24,249,676	24,249,689	24,250,629	24,251,501	24,252,474	24,252,561	24,252,603	24,252,664	24,254,607	24,255,286	24,255,455	24,257,371
BCAT	BCAT	BCAT	BCAT	BCAT	BCAT	BCAT	BCAT	BCAT	BCAT	BCAT	BCAT	BCAT	BCAT	BCAT	BCAT	BCAT				
C	G	T	A	G	T	A	C	C	C	C	C	T	-	T	A	G	T	A	A	A
C	G	T	A	G	T	A	C	C	C	C	C	T	-	T	A	G	T	A	A	A
C	G	T	A	G	T	A	C	C	C	C	C	T	-	T	A	G	T	A	A	A
C	G	T	A	G	T	A	C	C	C	C	C	T	-	T	A	G	T	A	A	A
C	G	T	A	G	T	A	C	C	C	C	C	T	-	T	A	G	T	A	A	A
C	G	T	A	G	T	A	C	C	C	C	C	T	-	T	A	G	T	A	A	A
C	G	T	A	G	T	A	C	C	C	C	C	T	-	T	A	G	T	A	A	A
C	G	T	A	G	T	A	C	C	C	C	C	T	-	T	A	G	T	A	A	A
C	G	T	A	G	T	A	C	C	C	C	C	T	-	T	A	G	T	A	A	A
C	G	T	A	G	T	A	C	C	C	C	C	T	-	T	A	G	T	A	A	A
C	G	T	A	A	T	A	C	C	C	C	C	T	A	T	G	A	A	A	G	A
C	G	T	A	A	T	C	C	C	C	C	C	T	A	T	G	A	A	A	G	G
C	G	T	A	A	T	C	C	C	C	C	C	T	A	T	G	A	A	A	G	G
C	G	T	A	A	C	C	C	C	A	G	C	T	A	T	G	A	A	A	G	G
C	G	T	A	A	T	C	G	C	A	G	T	T	A	T	G	A	A	A	G	G
C	G	T	A	A	T	C	C	C	C	C	C	T	A	T	G	A	A	A	G	G
A	T	C	G	G	C	A	G	T	A	G	T	C	A	G	G	A	T	G	G	G
A	T	C	G	G	C	A	G	T	A	G	T	C	A	G	G	A	T	G	G	G
A	T	C	G	G	C	A	G	T	A	G	T	C	A	G	G	A	T	G	G	G
A	T	C	G	G	C	A	G	T	A	G	T	C	A	G	G	A	T	G	G	G
A	T	C	G	G	C	A	G	T	A	G	T	C	A	G	G	A	T	G	G	G
C	G	T	A	A	T	C	C	C	C	C	C	T	A	T	G	A	A	A	G	G
C	G	T	A	A	T	C	C	C	C	C	C	T	A	T	G	A	A	A	G	G
C	G	T	A	A	T	C	C	C	C	C	C	T	A	T	G	A	A	A	G	G
C	G	T	A	A	T	C	C	C	C	C	C	T	A	T	G	A	A	A	G	G
C	G	T	A	A	T	C	C	C	C	C	C	T	A	T	G	A	A	A	G	G
C	G	T	A	A	T	C	C	C	C	C	C	T	A	T	G	A	A	A	G	G
C	G	T	A	A	T	A	C	C	C	C	C	T	A	T	G	A	T	A	G	G
C	G	T	A	A	T	C	G	C	A	G	T	T	A	T	G	A	A	A	G	G
C	G	T	A	A	T	C	C	C	C	C	C	T	A	T	G	A	A	A	G	G
C	G	T	A	A	T	C	C	C	C	C	C	T	A	T	G	A	A	A	G	G
C	G	T	A	A	T	A	G	C	A	G	T	T	A	T	G	A	T	A	G	G
A	T	C	G	G	C	A	G	T	A	G	T	C	A	G	G	A	T	G	G	G
A	T	C	G	G	C	A	G	T	A	G	T	C	A	G	G	A	T	G	G	G
A	T	C	G	A	C	C	G	T	A	G	T	C	A	G	G	A	A	G	G	G
A	T	C	G	G	C	A	G	T	A	G	T	C	A	G	G	A	T	G	G	G
A	T	C	G	G	C	A	G	T	A	G	T	C	A	G	G	A	T	G	G	G
C	G	T	A	A	T	C	C	C	C	C	C	T	A	T	G	A	A	A	G	G
C	G	T	A	A	T	C	C	C	C	C	C	T	-	T	G	A	A	A	G	G
C	G	T	A	A	T	C	C	C	C	C	C	T	A	T	G	A	A	A	G	G
C	G	T	A	A	T	C	C	C	C	C	C	T	-	T	G	A	A	A	G	G
C	G	T	A	A	T	C	C	C	C	C	C	T	A	T	A	A	T	A	G	G
C	G	T	A	A	T	A	C	C	C	C	C	T	A	T	A	A	T	A	G	A
C	G	T	A	A	T	C	C	C	C	C	C	T	A	T	G	A	A	A	G	G
C	G	T	A	A	T	C	C	C	C	C	C	T	A	T	G	A	A	A	G	G
C	G	T	A	A	T	C	C	C	C	C	C	T	A	T	G	A	A	A	G	G
C	G	T	A	A	T	C	C	C	C	C	C	T	A	T	G	A	A	A	G	G
C	G	T	A	A	T	C	C	C	C	C	C	T	A	T	G	A	A	A	G	G

Supplementary File 7 (Figure S4). Phylogenetic tree of 48 cucumber lines based on 68 SNPs and 3 indels within the 50-kb genomic region at the *F* locus. Details of the accessions, and markers are provided in Table S2.

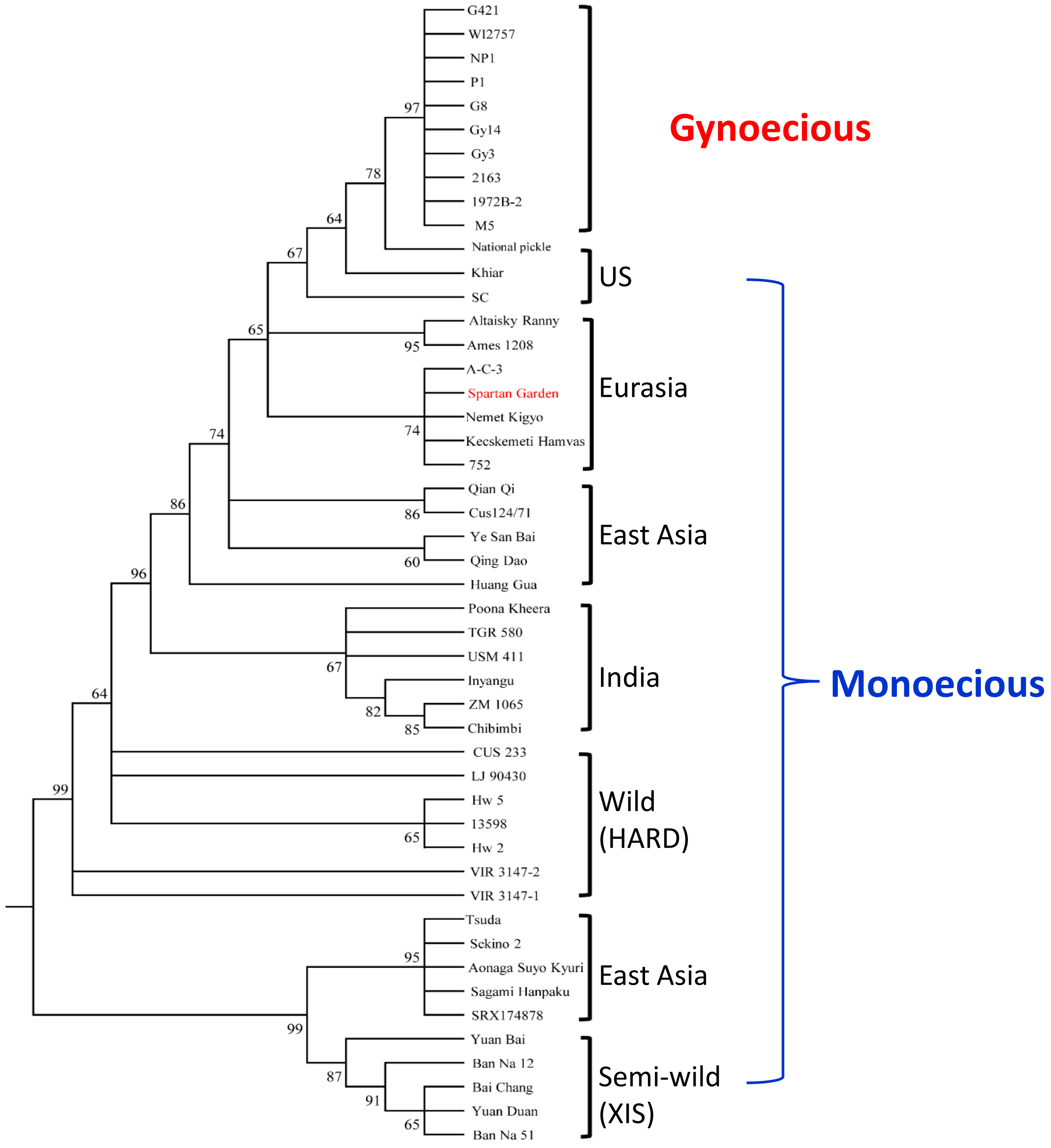

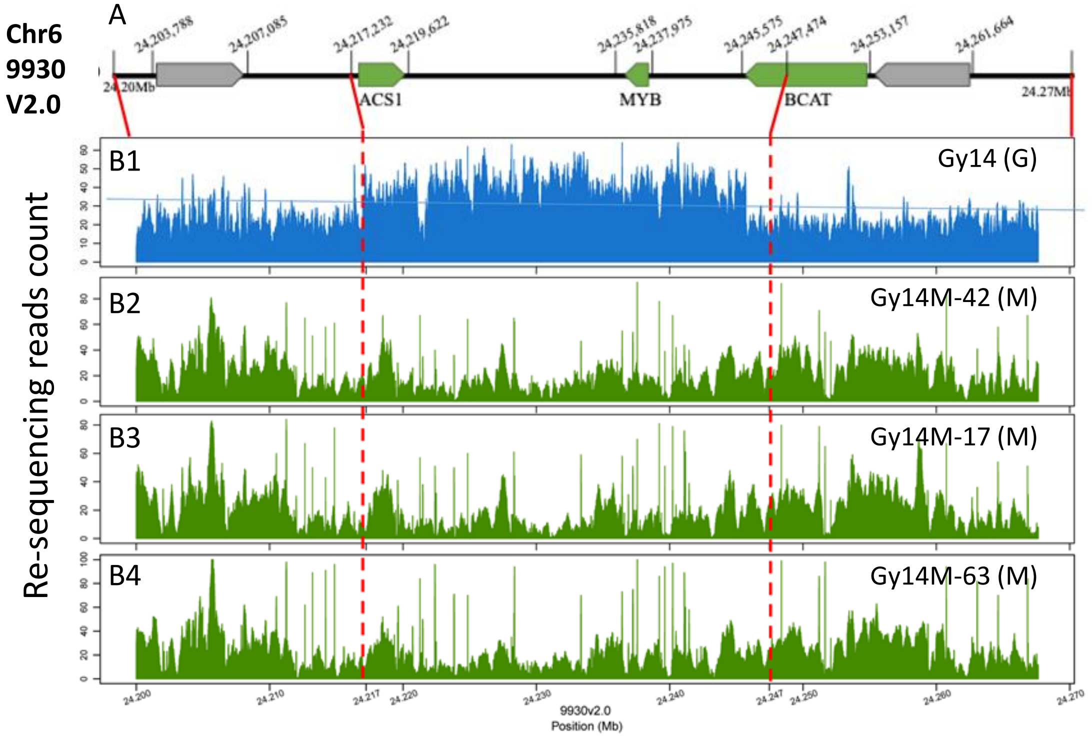

Supplemental File 8 (Figure S5). Copy number variation at this *F* locus in 'gynoecy loss' cucumber mutants. This region has ~30 kb in the monoecious line 9930 with three predicted genes (top, coordinates based on V2.0). Alignment of re-sequencing reads against the 30 kb region of 9930 reveals loss of the extra copy in the three monoecious mutants (B2-B4) as compared to their parental line Gy14 (B1). Vertical red dotted lines delimit the CNV region.

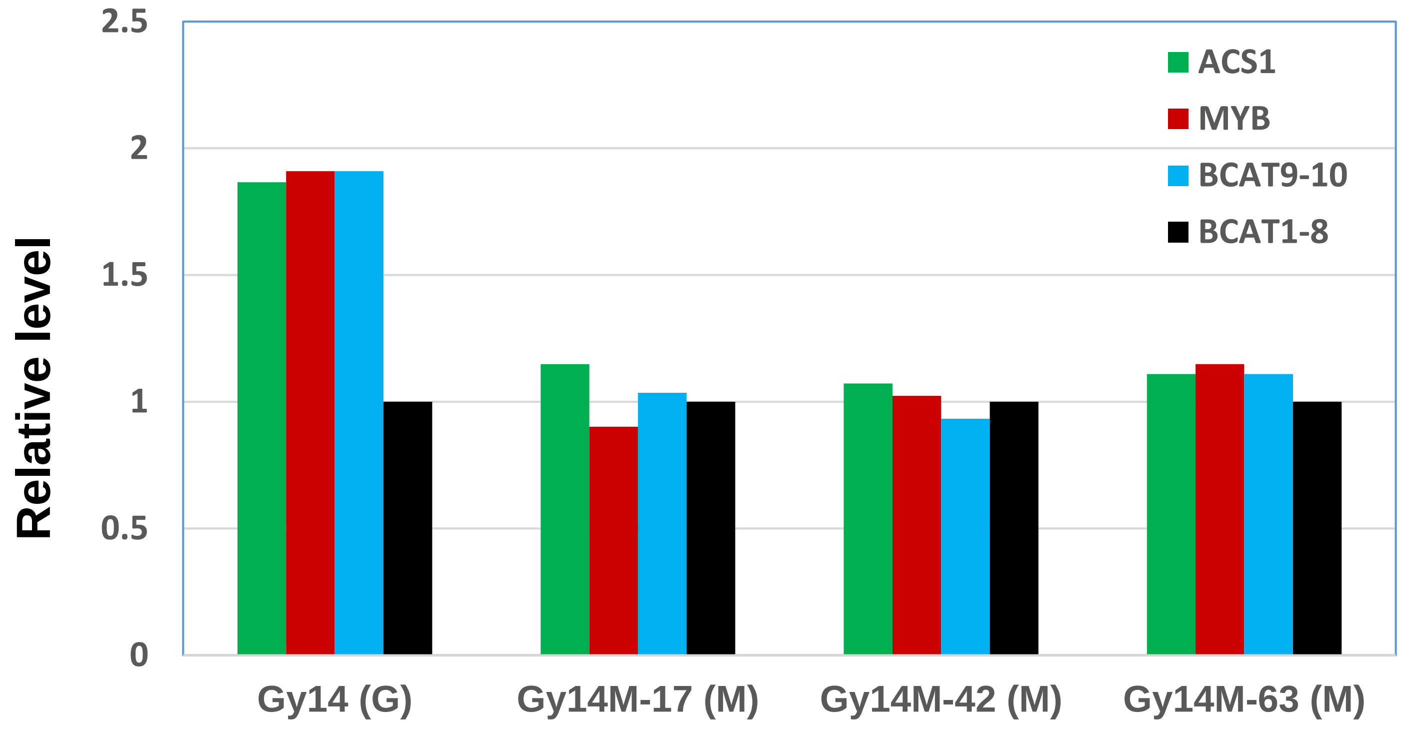

Supplementary File 9 (Figure S6). Copy numbers of different regions revealed by qPCR among Gy14 and the three ‘gynoecy-loss’ (Gy14M-17/42/63) lines. The detailed assay is according to Fig. 3B and C.

Supplementary File 10 (Figure S7). Sex expression of the F_1 plants from Gy14 × 9930 (A and B), Gy14M-17 x 9930 (C), and Gy14M42 x 9930 crosses (D). Plants in A and B are subgynoecious, and those in C and D are monoecious. Red and blue arrows indicate the male flowers in subgynoecious plants (A and B), and ovaries (female flowers) in monoecious plants (C and D), respectively.

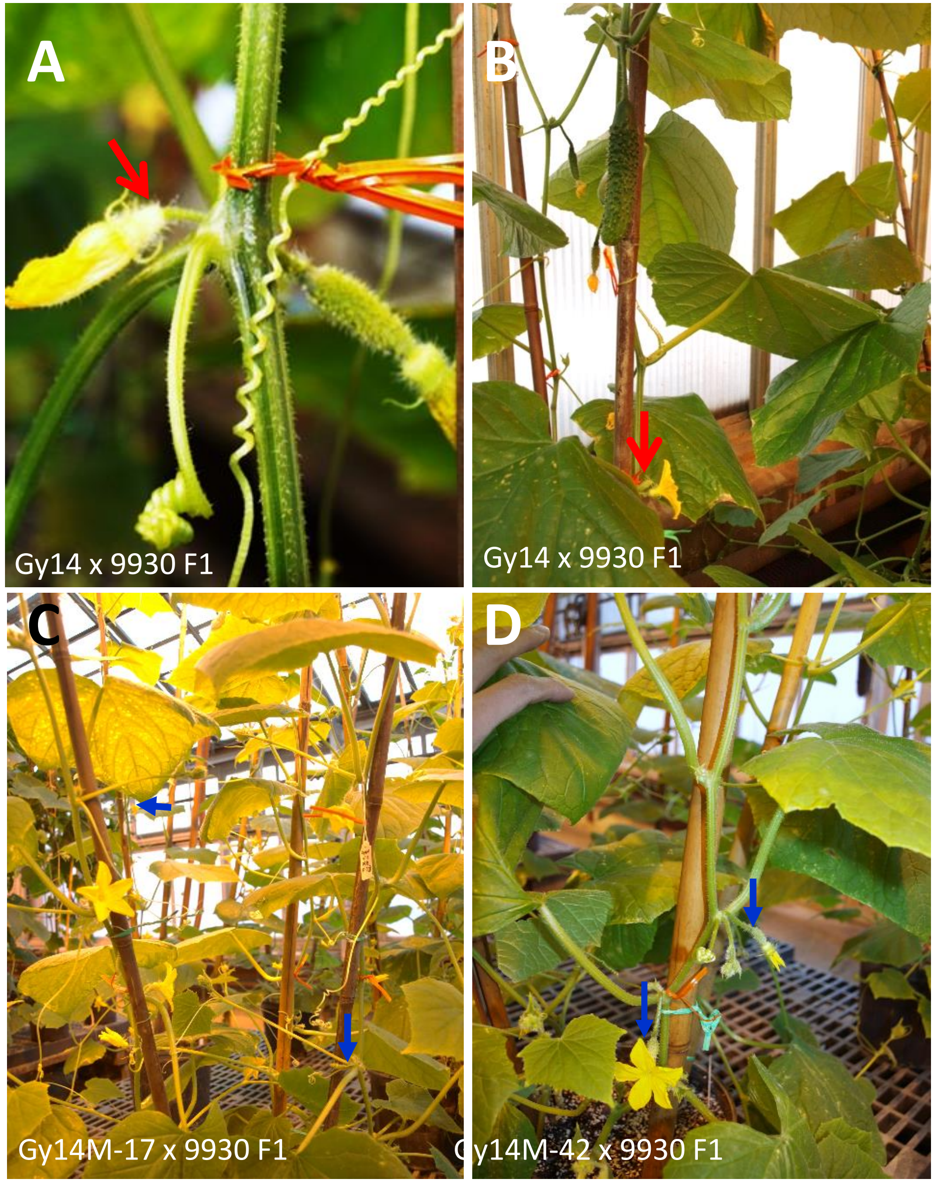

Supplement: Supplementary file 1 — Supplemental files [file 41438_2020_251_MOESM1_ESM.pdf]
